# Biomedical Importance of Indoles

**DOI:** 10.3390/molecules18066620

**Published:** 2013-06-06

**Authors:** Nagendra Kumar Kaushik, Neha Kaushik, Pankaj Attri, Naresh Kumar, Chung Hyeok Kim, Akhilesh Kumar Verma, Eun Ha Choi

**Affiliations:** 1 Plasma Bioscience Research Center, Kwangwoon University, Seoul 139701, Korea; E-Mails: attri.pankaj@gmail.com (P.A.); nash_bms@yahoo.co.in (N.K.); hyeokkim@kw.ac.kr (C.H.K.); 2 Department of Chemistry, University of Delhi, Delhi 110007, India

**Keywords:** indole, biomedical application, bioactivity, pharmacological activity

## Abstract

The indole nucleus is an important element of many natural and synthetic molecules with significant biological activity. This review covers some of the relevant and recent achievements in the biological, chemical and pharmacological activity of important indole derivatives in the areas of drug discovery and analysis.

## 1. Introduction

Heterocyclic chemistry is one of the most valuable sources of novel compounds with diverse biological activity, mainly because of the unique ability of the resulting compounds to mimic the structure of peptides and to bind reversibly to proteins [1,2,3,4]. To medicinal chemists, the true utility of heterocyclic structures is the ability to synthesize one library based on one core scaffold and to screen it against a variety of different receptors, yielding several active compounds. Almost unlimited combinations of fused heterocyclic structures can be designed, resulting in novel polycyclic frameworks with the most diverse physical, chemical and biological properties. The fusion of several rings lead to geometrically well-defined rigid polycyclic structures and, thus, holds the promise of a high functional specialization resulting from the ability to orient substituents in three dimensional space. Therefore, efficient methodologies resulting in polycyclic structures from biologically active heterocyclic templates are always of interest to both organic and medicinal chemists.

Compounds with heterocyclic rings are inextricably woven into the most basic biochemical processes of life. If one were to choose a step in a biochemical pathway at random there would be a very good chance that one of the reactants or products would be a heterocyclic compound. Even if this was not true, participation of heterocycles in the reaction in question would almost be certain as all biochemical transformations are catalyzed by enzymes, and three of the twenty amino acids found in enzymes contain heterocyclic rings. Of these, the imidazole ring of histidine in particular would be likely to be involved; histidine is present at the active sites of many enzymes and usually functions as a general acid-base or as a metal ion ligand. Furthermore, many enzymes function only in the presence of certain small non–amino acid molecules called coenzymes (or cofactor), which more often than not are heterocyclic compounds. But even if the enzyme in question contained none of these coenzymes or the three amino acids referred to above, an essential role would still be played by heterocycles as all enzymes are synthesized according to the code in DNA, which of course is defined by the sequence of the heterocyclic bases found in DNA.

Chemotherapy concerns the treatment of infectious, parasitic or malignant diseases by chemical agents, usually substances that show selective toxicity towards the pathogen. The diseases of bodily dysfunction and the agents employed are mainly compounds that affect the functioning of enzymes, the transmission of nerve impulses or the action of hormones on receptors. Heterocyclic compounds are used for all these purposes because they have a specific chemical reactivity for example epoxides, aziridines and *β*-lactams, because they resemble essential metabolites and can provide false synthons in biosynthetic processes, for example antimetabolites used in the treatment of cancer and virus diseases because they fit biological receptors and block their normal working, or because they provide convenient building blocks to which biologically active substituents can be attached. The introduction of heterocyclic groups into drugs may affect their physical properties, for example the dissociation constants of sulfa drugs, or modify their patterns of absorption, metabolism or toxicity.

Many significant discoveries have been made, however, by the rational development of observation of biological activity made by chance in work designed for other ends, or during the clinical use of drugs introduced for other purposes. The theoretical basis of medicinal chemistry has become much more sophisticated, but is naïve to suppose that the discovery of drugs is merely a matter of structure-activity relationships. The success of a medicinal agent depends on the balance between its desirable pharmacological effects and the harm it may otherwise do to a patient, and this cannot yet be predicated with certainty. Serendipity and luck will doubtless continue to play an important part in new discoveries.

## 2. Indole: Chemical and Biological Importance

**Figure 1:**
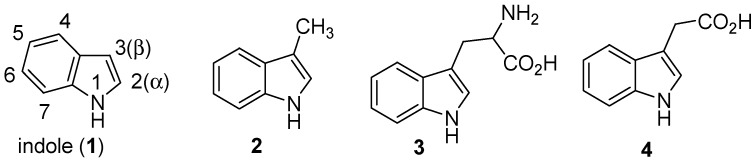
Indoles.

Indole (**1**, Figure 1) is the parent substance of a large number of important compounds that occur in nature. Indole and the simple alkylindoles are colourless crystalline solids with a range of odours from naphthalene-like in the case of indole itself to faecal in the case of skatole (3-methylindole, **2**) (Figure 1). Tryptophan [5] (2-amino-3-(3′-indolyl)propionic acid) (**3**, Figure 1) is one of the naturally occurring essential amino acids. Higher plants degrade tryptophan to heteroauxin (indole-3-acetic acid, **4**), a plant hormone (Figure 1).

**Figure 2:**
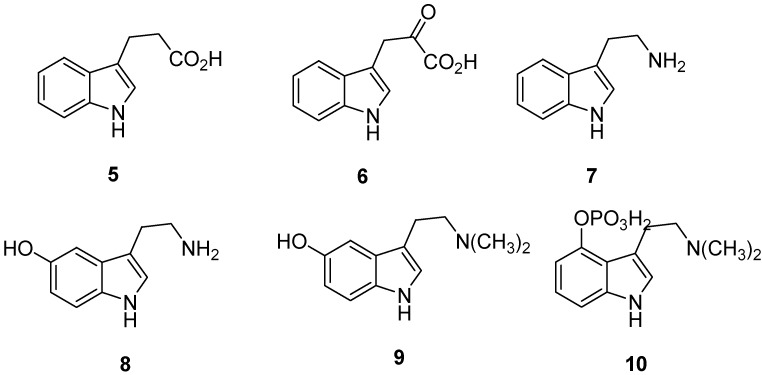
Derivatives of indoles.

The compounds 3-(3′-indoyl) propionic acid (**5**), indole-3-pyruvic acid (**6**), and the 1-, 2-, and 5-methylindole-3-acetic acids possess similar activity (Figure 2).

Bacteria degrade tryptophan to tryptamine [6] (2-(3′-indoyl)ethylamine) (**7**, Figure 2), which is the basis for some of the condensed ring alkaloids. Indole compounds that carry substituents, especially a hydroxy group, on the benzene ring include serotonin [7] (**8**, Figure 2) which is a vasoconstrictor hormone that plays a part in conducting impulses to the brain. Bufotenine (**9**, Figure 2) is found in the skins of toads, toxic mushrooms, and West Indian snuff, psilocybin (**10**, Figure 2) occurs in certain mushrooms. Both are known for their psychotropic effects [8].

**Figure 3:**
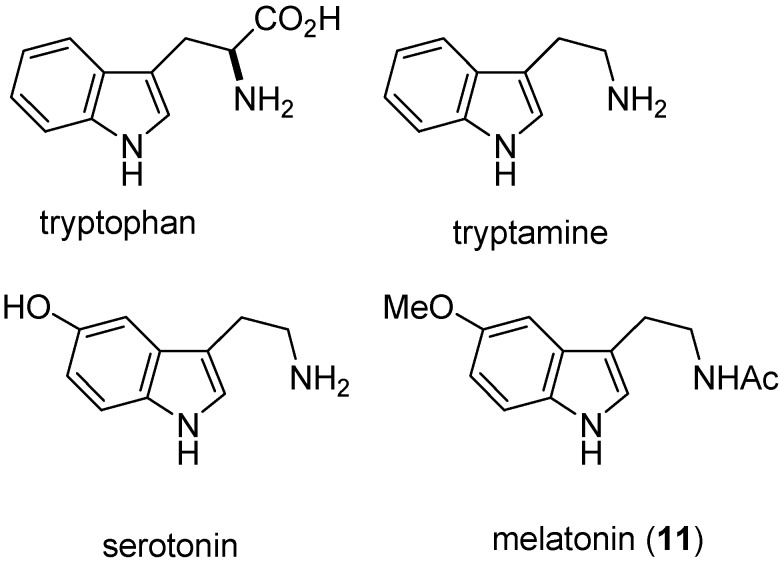
Structures of some naturally occurring indoles.

Some indole alkaloids exert considerable pharmacological activity but quite different effects may be obtained even from alkaloids of one genus, e.g., the Strychnos alkaloid strychnine acts powerfully causing muscle contraction, while the toxiferines act as muscle relaxants. Of the clinically useful alkaloids, three groups are notable: (a) the Ergot alkaloids—ergometrine with its direct action on the contraction of uterine muscle, ergotamine for migraine relief and the modified alkaloid, bromocriptine, which suppresses lactation and has some application for the treatment of mammary carcinoma; (b) the Rauvolfia alkaloids, and specifically reserpine, which was the forerunner of the tranquillisers; (c) the dimeric anti-leukemic alkaloids of *Catharanthus*, vinblastine and vincristine. One of the most exciting discoveries within the field of indole alkaloids has been the recognition of the role played by iridoid precursors such as secologanin. These discoveries have enabled the many diverse structures to be rationalized on the basis of an understanding of their biosynthesis. Indoles are probably the most widely distributed heterocyclic compounds in nature having medicinal importance (Figure 3). Tryptophan is an essential amino acid and as such is a constituent of most proteins; it also serves as a biosynthetic precursor for a wide variety of tryptamine-indole, and 2,3-dihydroindole-containing secondary metabolites. In animals, serotonin (5-hydroxytrytamine) is a very important neurotransmitter in the CNS, and also in the cardiovascular and gastrointestinal systems. The structurally similar hormone melationin (**11**) is thought to control the diurnal rhythm of physiological functions.

**Figure 4:**
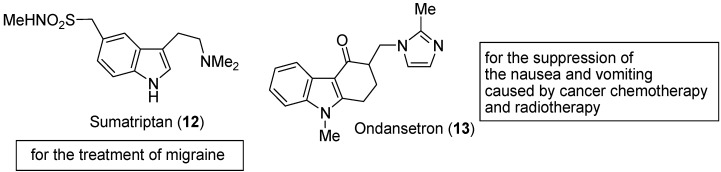
Structure of indoles used in chemotherapy.

Study and classification of 5-hydroxytryptamine receptors has resulted in the design and synthesis of highly selective medicines such as sumatriptan [9] (**12**) for the treatment of migraine, ondansetron [10] (**13**) for the suppression of the nausea and vomiting caused by cancer chemotherapy and radiotherapy (Figure 4), and alosetron [11] (**14**) for the treatment of irritable bowel syndrome.

**Figure 5:**
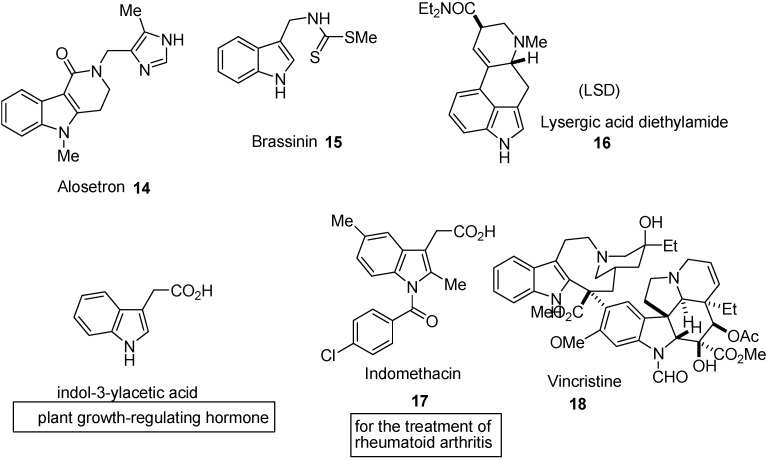
Indoles with important activity in plants and animals.

Tryptophan-derived substances in the plant kingdom include indole-3-ylacetic acid, a plant growth-regulating hormone, and a huge number and structural; variety of secondary metabolites ‒ the indole alkaloids [12] (Figure 5). In the past, the potent physiological properties of many of these led to their use in medicines, but in most instances these have now be supplemented by synthetic substances, although vincristine, a “dimeric” indole alkaloid is still extremely important in the treatment of leukemia. Brassinin [13] (**15**) isolated from turnips, is a “phytoalexin”-one of a group of compounds produced by plants as a defence mechanism against attack by microorganisms.

The physiological activity of lysergic acid diethylamide (LSD) is notorious. The synthetic indol-3-ylacetic acid derivative indomethacin is used for the treatment of rheumatoid arthritis. LSD (**16**) has been used in psychiatry for its perceived therapeutic value, in the treatment of alcoholism, pain and cluster headache relief, for spiritual purposes, and to enhance creativity [14]. Indomethacin (**17**) is a non-steroidal anti-inflammatory drug commonly used to reduce fever, pain, stiffness, and swelling. It works by inhibiting the production of prostaglandins [15].

A number of tubulin polymerization inhibitors characterized by the presence of an indole nucleus have been obtained from natural sources or have been prepared by semi-synthesis. Vincristine and vinblastine are among the earliest anti-tumor agents being recognized since 1,965 as tubulin polymerization inhibitors. These drugs remain of clinical interest. Vincristine [16] (**18**) is anti-tumor agents being recognized tubulin polymerization inhibitors and used in combination in the treatment of acute lymphoblastic leukemia and against both Hodgkin’s and non- Hodgkin lymphoma. Vinblastine is mainly used in the clinical treatment of advanced Hodgkin’s disease against germ cell cancer of the testes [17]. Many efforts have been taken aiming at the identification of novel, more active, and less cytotoxic semi-synthetic Vinca alkaloids. Among the large number of derivatives synthesized by academic or industrial groups, two semi-synthetic derivatives, vindesine and vinorelbine have been employed in anti-cancer therapy [17].

**Figure 6:**
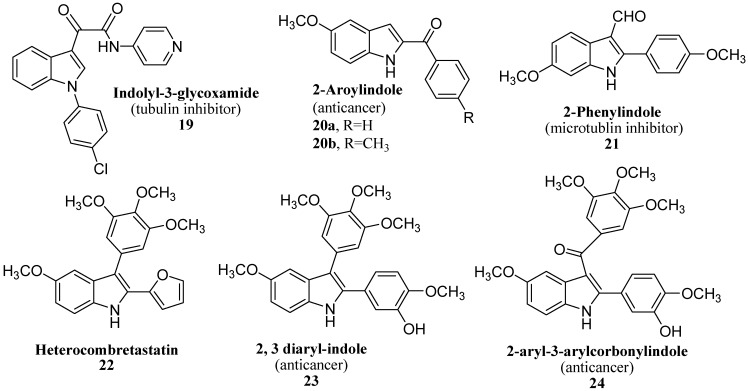
Indole derivatives as tubulin inhibitors.

The indole nucleus is the core structure of a great number of tubulin polymerization inhibitors (Figure 6) [18,19]. The indolyl-3-glyoxamide D-24851 (**19**) and the 2-aroylindoles D-64131 (**20a**) and D-68144 (**20b**) were discovered by Baxter Oncology. Compounds **20** are highly active against various tumors, including those resistant to paclitaxel [20]. Several 2-phenylindoles were designed by von Angerer as simple analogues of 12-formyl-5,6-dihydroindolo[2,1-*a*]isoquinoline. Among them, indole 2-phenylindole **21** completely blocked microtubule assembly at a concentration of 40 µM [21]. On the basis of the structure of the natural product combretastatin A-4 (CSA4), some 2,3-diarylindoles, known as heterocombretastatins **22**, were prepared by Medarde [22]. Flynn *et al*. reported the tubulin polymerization inhibitory activity of 2,3-diarylindoles **23** and 2-aryl-3-arylcarbonylindoles **24** [23]. Sulfur containing compounds, such as sulfonamide E-7010 [24], thiophene [25] and benzothiophene [23] derivatives, proved effective inhibitors of tubulin polymerization. To our knowledge there have been no reports on the inhibition of tubulin polymerization by arylthio/sulfonylindoles.

**Figure 7:**
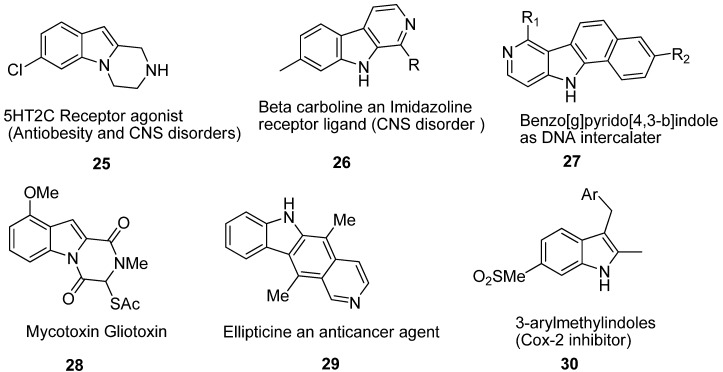
Pharmacologically active indole derivatives.

In this race to synthesize new drugs, indoles have attracted a great deal of attention amongst the scientific community due to their therapeutic uses (Figure 7). Researchers from Roche and Vernalis, respectively, described the discovery of 5-hydroxytryptamine 2c (5-HT_2c_) agonist **25** based on the pyrazino[1,2-*a*]indole scaffold [26]. Indeed selectivity is one of the most important points in the design of 5-HT_2c_ agonists, as cardiovascular and psychotomimetic effects have been described these compounds. It recently has been shown that *β*-carbolines **26** represent a novel class of imidazoline-2 (I_2_) ligands lacking an imidazoline moiety [27]. For example, harmane, norharmane and 1,2,3,4-tetrahydro-β-carboline (THBC) bind at I_2_ receptors with high affinity [28] (*K*_i_ = 49 nM, 87 nM, and 9.4 nM, respectively). A preliminary structure-affinity study has been conducted; the fully aromatic compounds harmane and norharmane displayed <10-fold) selectivity for I_2_ versus I_1_ binding sites, but THBC displayed >1,000-fold I_2_ selectivity. Some compounds of the benzopyrido[4,3-β] indole class **27** are also reported as DNA intercalaters [29].

Pyrazino[1,2-*a*]indole-1,4-diones , as simple analogues of gliotoxin (**28**, Figure 7) act as selective inhibitors of geranylgeranyltransferase I. Gliotoxin is a natural epidithiodiketopiperazine mycotoxin with immunosuppressive and antimicrobial activity. Gliotoxin and the related fungal metabolites gliovirin and sporedesmin are low molecular weight non-polar compounds characterised by an intramolecular disulfide bridge that is the active moiety [29,30,31]. It has been observed that gliotoxin inhibits farnesyltransferase (FTase) at low micromolar concentrations [32,33,34], and has antiproliferative activity in lymphosarcoma cells. Ellipticine (**29**, Figure 7) is an antineoplastic agent, the mode of action of which was considered to be based mainly on DNA intercalation and/or inhibition of topoisomerase II [35,36]. Researchers showed that ellipticine also covalently binds to DNA after being enzymatically activated [35,37,38,39]. Using human recombinant cytochrome P450 (CYP) enzymes, CYP3A4, 1A1, and 1B1–those enzymes expressed in tumors sensitive to ellipticine (*i.e.*, breast cancer) [40]—were found to be the most efficient CYPs activating ellipticine to form covalent DNA adducts [35]. Deoxyguanosine was identified to be the target for CYP-mediated ellipticine binding [39]. The formation of these adducts was also detected in V79 lung fibroblast cells transfected with human CYP3A4, 1A1, and 1A2, [38] in human breast adenocarcinoma MCF-7 cells [41], and *in vivo* in rats exposed to ellipticine [40]. On the basis of these data, ellipticine might be considered a drug for which pharmacological efficiency and/or genotoxic side effects are dependent on its enzymatic activation in target tissues [35,37,38,39,41]. For an anti-inflammatory program targeting potent and selective cyclooxygenase-2 (COX-2) inhibitors **30** (Figure 7), Campbell *et al*. described a mild general method for the one-pot 3-arylmethylation of indoles containing a 6-methylsulfonyl moiety [42].

## 3. Indole Ring Containing Important Marketed Drug Molecules

**Figure 8:**
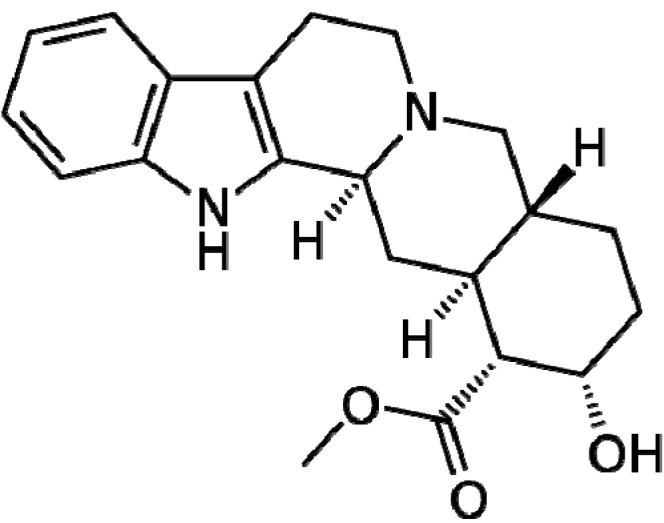
Yohimbine: Drug for male impotency.

**Figure 9:**
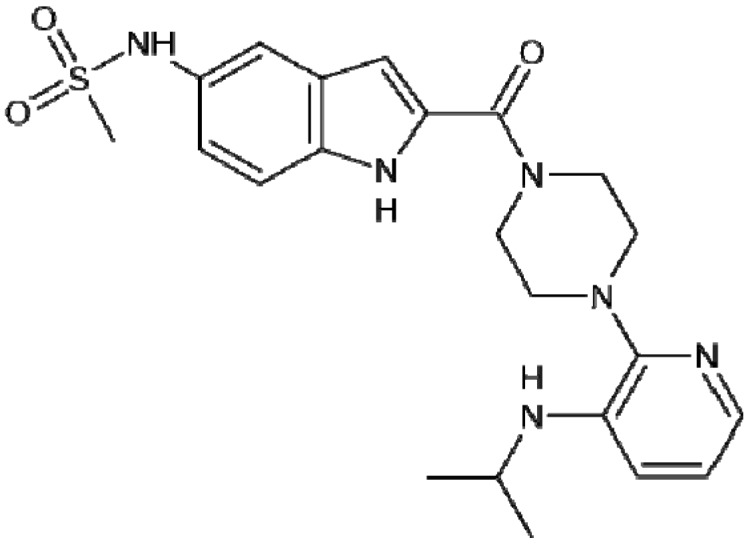
Delavirdine: Anti-HIV drug.

**Figure 10:**
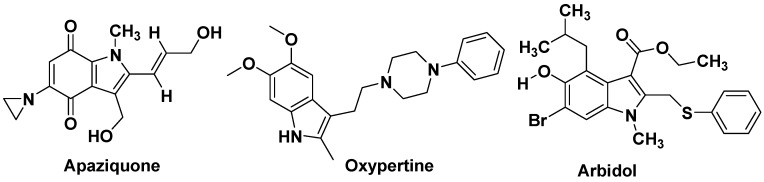
Apaziquone as anticancer, Oxypertine as antipsychotic and Arbidol as antiviral.

Table 1 lists important indole ring-containing marketed drugs and their associated biological activities. Recently, the indole ring-containing compound yohimbine (17α-hydroxyyohimban-16α-carboxylic acid methyl ester, Figure 8) was proved by researchers for the treatment of sexual dysfunction [43]. Yohimbine was also explored as a remedy for type-2 diabetes in animal and human models, carrying polymorphisms of the α2A-adrenergic receptor gene [44]. Delavirdine (Figure 9), an inhibitor of cytochrome P450 isozyme CYP3A4, is also a drug with an indole ring developed for the treatment of HIV type 1 [45]. The indole-based pharmaceutical constitute very important class of therapeutic molecules and are likely to replace many existing pharmaceuticals in the future [46]. The biological profiles of this new generation of indoles represent much progress with regard to the older compounds. Apaziquone (EOquin, Figure 10) is an indolequinone that is a prodrug and a chemical analog of the older mitomycin C. In a hypoxic environment, such as those on the inner surface of the urinary bladder, apaziquone is converted to active metabolites by intracellular reductases. The active metabolites alkylate DNA and lead to apoptotic cell death. This activity is preferentially expressed in neoplastic cells [47].

**Table 1:** Indole ring containing drug molecules.

*Drug*	*Application*	*Drug*	*Application*	*Drug*	*Application*
**Vincristine**	Anticancer	**Vincamine**	Vasodilator	**Roxindole**	Schizophrenia
**Vinblastine**	Anticancer	**Reserpine**	Antihypertensive	**Delavirdine**	Anti-HIV
**Vinorelbine**	Anticancer	**Peridopril**	Antihypertensive	**Atevirdine**	Anti-HIV
**Vindesine**	Anticancer	**Pindolol**	Antihypertensive	**Arbidol**	Antiviral
**Mitraphylline**	Anticancer	**Binedaline**	Antidepressant	**Zafirlukast**	Anti-Asthmatic
**Cediranib**	Anticancer	**Amedalin**	Antidepressant	**Bucindolol**	β-Blockers
**Panobinostat**	Anti-leukamic	**Oxypertine**	Antipsychotic	**Pericine**	Opioid agonist
**Apaziquone**	Anticancer	**Siramesine**	Antidepressant	**Mitragynine**	Opioid agonist
**Tropisetron**	Antiemetic	**Indalpine**	Antidepressant	**Pravadoline**	Analgesic
**Doleasetron**	Antiemetic	**Yohimbine**	Sexual Disorder	**Bufotenidine**	Toxin
**Oglufanide**	Immunomodulatory	**Indomethacin**	Anti-inflammatory	**Proamanullin**	Toxin

Oxypertine (Figure 10) is an antipsychotic and antidepressant used in the treatment of schizophrenia. Chemically, it is an indole derivative similarly to molindone and a member of the phenylpiperazine class [48]. Arbidol (Figure 10) is an antiviral treatment for influenza infection used in Russia and China. The drug is manufactured by Pharmstandard and since 2005 it has been the number one best-selling over-the-counter drug in Russia. Chemically, arbidol features an indole core, functionalized at all positions but one with different substituents. The drug inhibits viral entry into target cells, and also stimulates the immune response [49]. Perindopril is one of the most prescribed inhibitors of angiotensin converting enzyme, has a large evidence base, which allows to use it in patients with hypertension, diabetes mellitus type 2, coronary heart disease and chronic heart failure. Researchers also showed many evidences of the organoprotective properties of perindopril [50].

**Figure 11:**
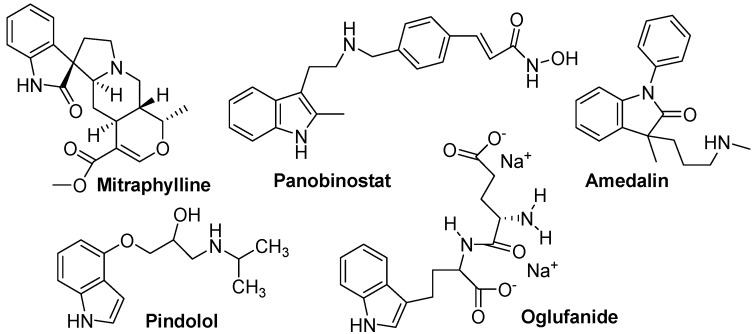
Mitraphylline as anticancer, panobinostat as antileukamic, amedalin as antidepressant, pindolol as antihypertensive, oglufanide as Immunomodulator.

**Figure 12:**
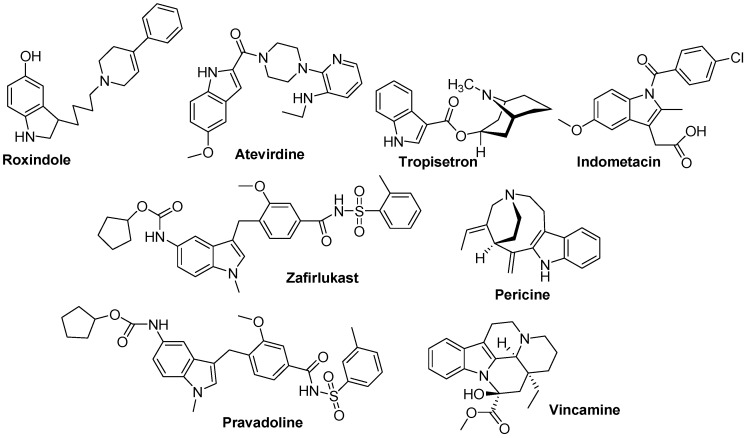
Important indole ring-containing drugs.

Mitraphylline (Figure 11), an oxindole derivative, is an active alkaloid found in the leaves of the tree *Mitragyna speciosa*, commonly known as kratom. Current research on mitraphylline is focussing on its antiproliferative effects and its *in-vivo* efficacy to induce apoptosis in human breast cancer, sarcoma and leukaemia cell lines [51,52]. Panobinostat (Figure 11) is also a drug developed by Novartis for the treatment of various cancers. Panobinostat was tested against Hodgkin's Lymphoma, cutaneous T cell lymphoma and other types of malignant disease in Phase III clinical trials, against myelodysplastic syndromes, breast cancer and prostate cancer in Phase II trials, and against chronic myelomonocytic leukemia in a Phase I trial [53,54]. Amedalin (Figure 11) is an antidepressant which was synthesized in the early 1970s, It is a selective norepinephrine reuptake inhibitor, but was never marketed. Pinodolol (Figure 11) is a beta blocker that is added to standard antidepressant therapy, if the patient fails to respond to the standard therapy alone [55]. Oglufanide (Figure 11), at one time called thymogen, is a dipeptide isolated from calf thymus. The immunomodulatory properties of both the natural product oglufanide and the subsequent synthetic versions of oglufanide have been extensively studied as an agent that enhances the immune function. The compound is currently undergoing clinical trials in patients infected with the hepatitis C virus [56]. Roxindole (Figure 12) was originally developed for the treatment of schizophrenia. In clinical trials its antipsychotic efficacy was only modest, but it was unexpectedly found to produce potent and rapid antidepressant and anxiolytic effects too. It has also been investigated as a therapy for Parkinson's disease and prolactinoma [57]. Tropisetron is a serotonin 5-HT_3_ receptor antagonist used mainly as an antiemetic to treat nausea and vomiting following chemotherapy, although it has been used experimentally as an analgesic in cases of fibromyalgia [58]. Tropisetron (Figure 12) acts as both a selective 5-HT_3_ receptor antagonist and α7-nicotinic receptor agonist [59,60]. Ateviridine (Figure 12) is non-nucleoside reverse transcriptase inhibitor that has been studied for the treatment of HIV [61]. Indometacin or indomethacin (Figure 12) is a non-steroidal anti-inflammatory drug (NSAID) commonly used as a prescription medication to reduce fever, pain, stiffness, and swelling. It works by inhibiting the production of prostaglandins, molecules known to cause these symptoms [15,62,63]. Indometacin is marketed under more than seventy different trade names [64]. Zafirlukast (Figure 12) is an oral leukotriene receptor antagonist (LTRA) for the maintenance treatment of asthma, often used in conjunction with an inhaled steroid and/or long-acting bronchodilator. Pericine (Figure 12) is one of a number of indole alkaloids found in the tree *Picralima nitida*, commonly known as Akuamma. The dried seeds of *Picralima nitida* used in traditional medicine throughout West Africa, particularly in Ghana as well as in the Ivory Coast and Nigeria. Pericine has been shown to bind to opioid receptors, and has an IC_50_ of 0.6 μmol, around 6 times more potent than codeine [65,66]. Pravadoline (Figure 12) was found to exhibit unexpectedly strong analgesic effects, which appeared at doses ten times smaller than the effective anti-inflammatory dose and so could not be explained by its action as a COX inhibitor. These effects were not blocked by opioid antagonists such as naloxone, and it was eventually discovered that pravadoline represented the first compound from a novel class of cannabinoid agonists, the aminoalkylindoles [67,68]. Vincamine (sold under the trademark Oxybral-SR, Figure 12) is a peripheral vasodilator that increases blood flow to the brain can be used as a nootropic agent to combat the effects of aging. Vincamine is an indole alkaloid found in the *Vinca minor* and *Catharanthus roseus* [69]. Vincamine can be synthesized in the lab from related alkaloids [70].

## 4. Focus on Bioactive Indoles Developed over the Past Few Years

### 4.1. Natural Products Containing an Indole Core Nucleus

**Figure 13:**
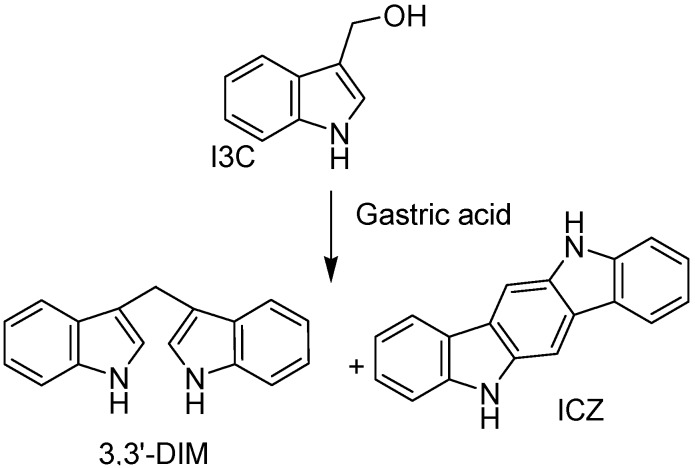
I3C and DIM.

Some plants and fungi are rich in indole-containing molecules, include indole-3-carbinol, harmane, lysergic acid, bufotenin, serotonin, tryptamine. In contrast, the anticancer potential of indole derivatives present in these vegetables is still largely unknown. Indole 3-carbinol (I3C; CAS No. 700-06-1) is a key bioactive molecule of cruciferous vegetables and well explored for prevention of few type of cancers (colorectal, lymphoma, breast, trans-placental cancer in offspring and prostate cancer) [71,72,73,74,75,76,77]. Ingested I3C can be converted into a biologically active dimer, 3,3′-diindolylmethane (DIM), within the gastrointestinal tract. Since DIM accumulates in the cell nucleus, it likely contributes to cell nuclear events that have been ascribed to I3C. Several mechanisms may account for the anticancer properties of I3C/DIM including changes in cell cycle progression, apoptosis, carcinogen bioactivation and DNA repair [78,79] (Figure 13).

**Figure 14:**
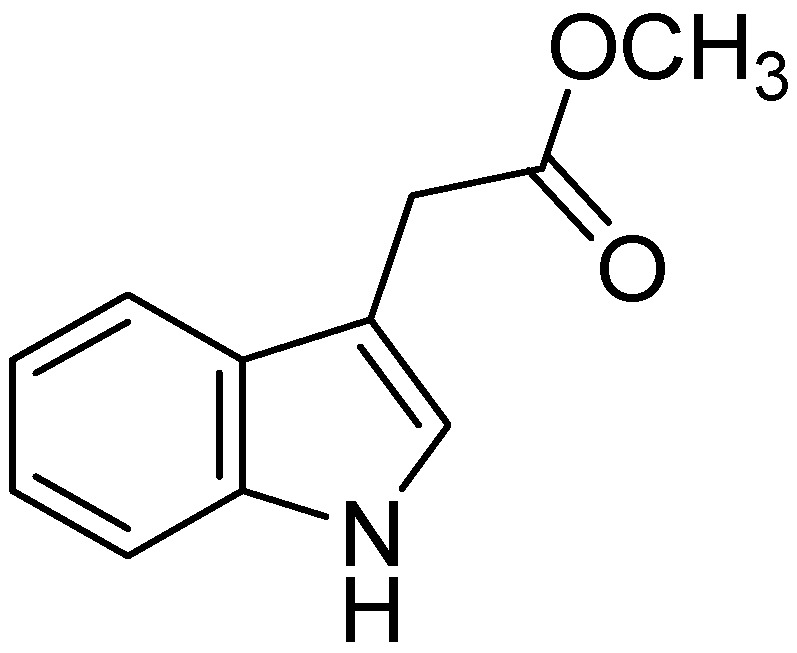
Methyl-3-indolyacetate (MIA).

Recently, I3C was also reported to have anti-inflammatory effects by inhibiting the production of NO, TNF-, and IL-10, in lipopolysaccharide (LPS)-stimulated RAW 264.7 macrophages [80]. Methyl-3-indolyacetate (MIA, Figure 14) found in cruciferous vegetables, also inhibits cancer cell invasion by targeting the MEK1/2-ERK1/2 signaling pathway. Recently, researchers observed that methyl-3-indolyl acetate, an indole derivative, was present in a large variety of cruciferous vegetables, including cabbage, broccoli, Brussels sprout, mustard, Thai kale, *etc*., at concentrations ranging from 20 to 100 μg/g (dried weight of vegetables) [81]. Overall recent research suggests that both dietary broccoli or cruciferous vegetables and the individual component indole-3-carbinol may offer protection from a far broader array of diseases than cancer, including cardiovascular and neurodegenerative diseases. A common link between these oxidative degenerative diseases and cancer may be aggravation by inflammation. Components of broccoli may protect against inflammation, inhibiting cytokine production. It remains to be seen whether cancer, cardiovascular disease, dementia and other diseases of aging can all benefit from a diet rich in broccoli and other crucifers [82]. Recently, Shin *et al*. evaluated the anti-inflammatory potential of the indole-containing fraction from the roots of *Brassica rapa* (IBR) (family Brassicaceae) and the underlying mechanisms. Their data suggest that the expressional inhibitions of iNOS, TNF-α, and IL-6 caused by an attenuation of NF-κB activation are responsible for the anti-inflammatory and antinociceptive activity of IBR [83].

Four indole alkaloids from *Catharanthus roseus* (Apocynaceae), a medicinal plant that produces more than 130 alkaloids, were identified (catharanthine, ajmalicine, tabersonine and lochnericine). Researchers evaluated the cytotoxic activity of the indole alkaloid-enriched bioactive extract obtained from suspension cultured-cells of *C. roseus* elicited with methyl jasmonate (MJ) and cyclodextrins (CDs) in three cell lines: JURKAT E.6 human lymphocytic leukemia, THP-1 human monocytic leukemia and BL 1395 non-tumor human B-cell line. The concentration of the indole alkaloid-enriched bioactive extract that inhibited cell growth by 50% (IC_50_) was 211–210 ng/mL for the blood cancer cell lines JURKAT E.6 human lymphocytic leukemia, THP-1 human monocytic leukemia [84]. The results confirm that the powerful anticancer activity of this indole alkaloid-enriched bioactive extract is not due to the effect of a single compound, but rather depends on the synergistic action of the four identified compounds [84].

**Figure 15:**
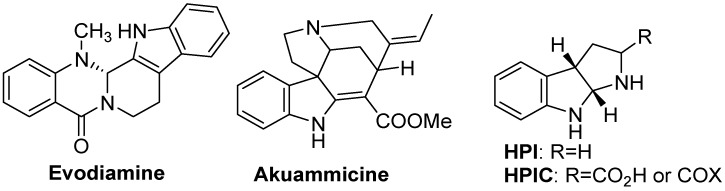
Evodiamine as multipurpose herbal medicine, akummicine for diabetes, HPI and HPIC.

Evodiamine (Figure 15), a naturally occurring indole alkaloid, is one of the main bioactive ingredients of Evodiae fructus. The fruit of *Evodiae fructus*, which also called “Wu-Zhu-Yu” (in Chinese) is one of the most popular and multipurpose herbs traditionally used in China for the treatment of headaches, abdominal pain, difficult menstruation, vomiting, diarrhea, and other disorders. Evodiamine is active against various disorders, including cancer, obesity, nociception, inflammation, cardiovascular diseases, Alzheimer’s disease, infectious diseases and it has themoregulative effects. Evodiamine can be used as a promising scaffold for the development of a novel class of multi-target-directed drug molecules, which can be used for various kinds of disorders or diseases [85]. Recently researchers reported that akuammicine increased glucose uptake in fully differentiated 3T3-L1 adipocytes after 24 h incubation. Akuammicine (Figure 15), an indole alkaloid, isolated from the chloroform extract of the seeds of *Picralima nitida* (Apocynaceae) stimulated glucose uptake in differentiated adipocytes, an activity related to the use of the seeds of *P. nitida* in the management of diabetes mellitus-II [86]. More recently, the hexahydropyrrolo[2,3-*b*]indole (HPI) unit, or the corresponding 2-carboxylate or 2-carboxamide (both abbreviated HPIC, Figure 15) containing natural product molecules are also showed wide range of biological activities, encompassing acyl-CoA inhibitors, neuropeptide neurotransmitter antagonists, topoisomerase inhibitors, and antibiotics [87].

### 4.2. Marine Product Containing Indole Core Nucleus

Marine natural products offer an abundant source of pharmacologically active agents with great diversity and complexity, and the potential to produce valuable therapeutic entities. A great variety of simple and substituted indole derivative, including halogenated indoles, bisindoles,and tryptamine derivatives have been previously isolated from marine organisms, with wide occurrence amongst variety of marine sources such as sponges, tunicates, algae, worms and microorganisms and have been extensively studied for their biological activities. Some marine organism also produce indole based bioactive secondary metabolites involved in their defence mechanisms. Marine organisms have confirmed to be a promising source of potentially valuable drugs against various human disorders or diseases.

**Figure 16:**
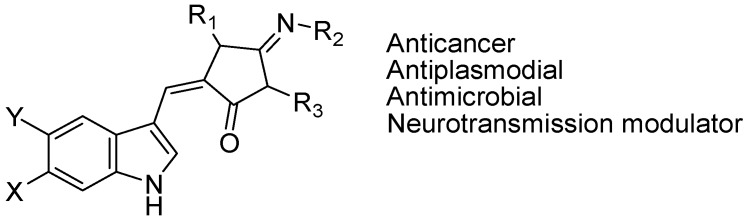
Aplysinopsin.

The aplysinopsins (Figure 16) are tryptophan-derived indole-containing marine natural products isolated from sponges, corals, an sea anemone and nudibranch. Aplysinopsins are widely distributed in the Pacific, Indonesia, Caribbean, and Mediterranean regions. Up to date, around 30 analogues occurring in Nature have been reported, however these aplysinopsin derivatives differ in chemical reactivity. The aplysinopsins have aroused considerable interest as potentially useful medicines. They have toxicity against many cancer cells and as well as anti-plasmodial and antimicrobial activity. They are also well known for modulation of neurotransmission, and have potential to influence monoaminooxidase (MAO) and nitric oxide synthase (NOS) activities. They can also act as serotonin receptors modulators [88]. 

**Figure 17:**
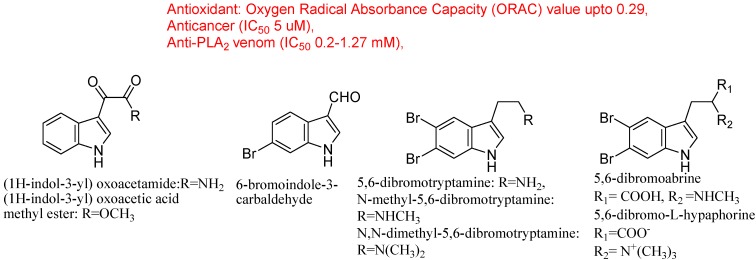
Indoles from sponges.

**Figure 18:**
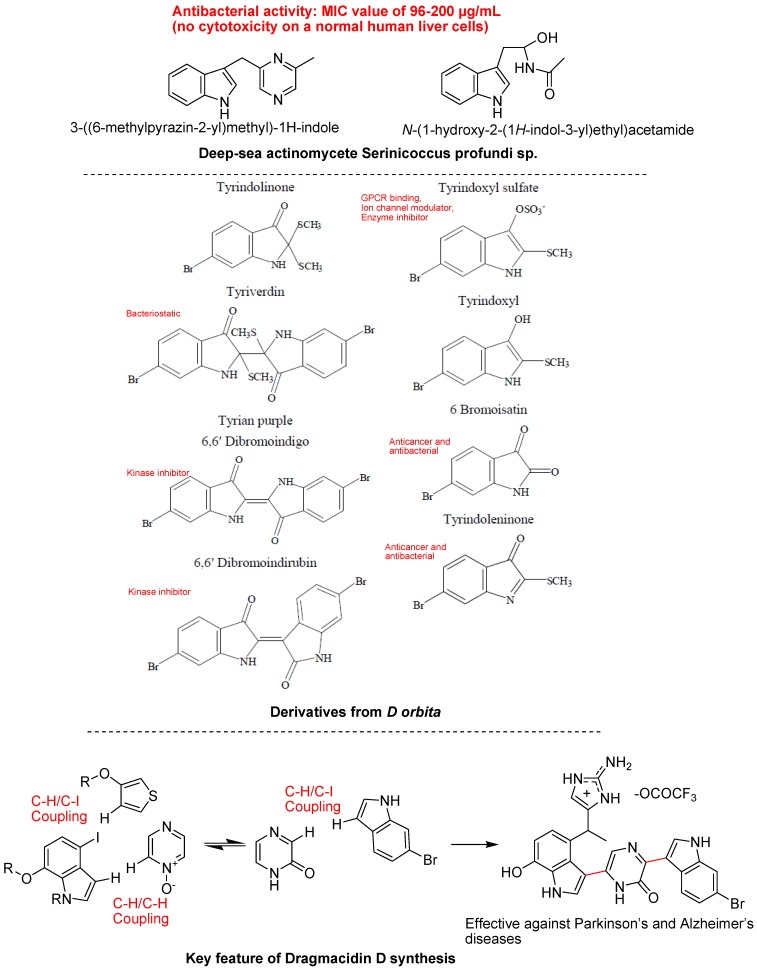
Indoles from deep sea species.

Many more indole derivatives, including bromoindoles, were isolated from the South Pacific marine sponges *Rhopaloeides odorabile* and *Hyrtios* sp. (Figure 17). Three known monomeric indoles were isolated from *R. odoabile* marine sponges {(1*H*-indol-3-yl) oxoacetamide, (1*H*-indol-3-yl) oxoacetic acid methyl ester and 6-bromoindole-3-carbaldehyde} and five dibromoindole derivatives (5,6-dibromotryptamine, *N*-methyl-5,6-dibromotryptamine, *N,N*-dimethyl-5,6-dibromotryptamine, and 5,6-dibromoabrine, 5,6-dibromo-L-hypaphorine), including the new derivative, 5,6-dibromo-L-hypaphorine were obtained from sponges (Figure 17). These derivatives could be promising in cosmetics due to their antioxidant activity and in pharmaceutics due to their anticancer, anti-inflammatory and anti-PLA2 potentials [89]. Among marine natural product chemical family, a sponge-derived bis-indole alkaloid fascaplysin also exhibited broad range of bioactivities including antibacterial, antifungal, antiviral, anti-HIV-1-RTase, p56 tyrosine kinase inhibition, antimalarial, anti-angiogenic, antiproliferative activity against numerous cancer cell lines, specific inhibition of cyclin-dependent kinase-4 and action as a DNA intercalator [90]. Many marine products contain brominated indolic rings such as 5,6-dibromotryptamine, 5,6-dibromo-*N*-methyltryptamine, 5,6-dibromo-*N*-methyltryptophan, 5,6-dibromo-*N*,*N*-dimethyltryptamine, 5,6-dibromo-L-hypaphorine, have shown anti-cancer, antimicrobial and anti-inflammatory properties. Potential use of these dibrominated indole metabolites in the treatment of depression-related pathologies was also suggested [91]. Recently, one new alkaloid, 3-((6-methylpyrazin-2-yl)methyl)-1*H*-indole, was obtained from the deep-sea actinomycete *Serinicoccus profundi* sp. nov., along with five known compounds (Figure 18). However, the new indole alkaloid displayed weak antimicrobial activity and no cytotoxicity on a normal human liver cell line (BEL7402) and a human liver tumor cell line (HL-7702) [92]. 

The predatory marine gastropod *Dicathais orbita* (Australian Muricidae) has been the subject of a significant amount of biological and chemical research over the past five decades [93,94,95]. Isolated brominated indoles and choline esters act as precursors of Tyrian purple, conform to Lipinski’s rule of five for drug-likeness and their predicted receptor binding, enzyme inhibitor activity and have a range of biological activities (Figure 18). The biological and chemical insight of *D. orbita* indoles provides a basis for future research in marine natural product chemistry [96]. Several bacterial cultures were isolated from a *Halichondria* sp. sponge collected from the Gujarat coast of the Indian Ocean region. The culture filtrate showed significant antimicrobial activity against 16 strains of clinical pathogens. The most potent antimicrobial compounds produced by *Bacillus licheniformis* SAB1 (GenBank accession number: DQ071568) were identified as indole, 3-phenylpropionic acid and a dimer 4,4′-oxybis[3-phenylpropionic acid. This significant antimicrobial activity may be due to their synergistic effect on microbial strains [97]. Recently, dragmacidine-D having 2 indole-azine bonds and an aminoimidazole unit, which was isolated from marine sponges, showed activity against Alzheimer’s, Parkinson’s and Huntington’s diseases. Prominent structural features of this compound are the two indole-pyrazinone bonds and the presence of a polar aminoimidazole unit. Recently researchers established a concise total synthesis of dragmacidin D using direct C–H coupling reactions; they constructed the core structure of dragmacidin D in a step-economical fashion (Figure 18) [98]. 

**Figure 19:**
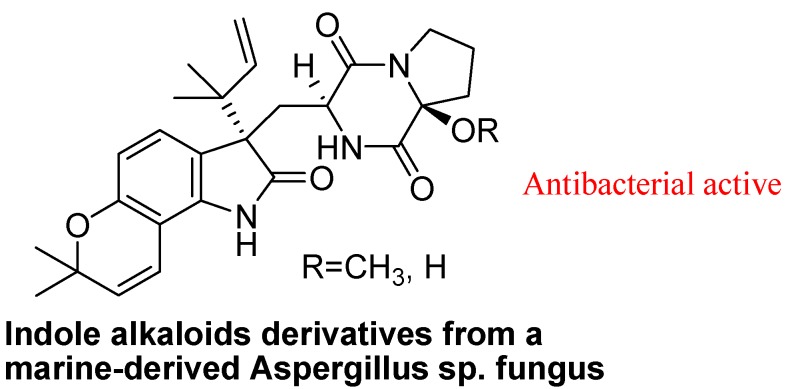
Indoles isolated from marine fungus

Marine fungi have also attracted increasing attention from those seeking new medicinal useful marine natural products in recent years. During the past few years, a growing number of biologically active natural compounds have been isolated from marine fungi. For example, the notoamides from *Aspergillus* spp. are prenylated indole alkaloids that incorporate complex bicycle(2.2.2)diazaoctane or diketopiperazine ring systems and show a wide range of biological activities. Recently, some new bioactive prenylated indole alkaloids were isolated from a marine-derived *Aspergillus* sp. fungus (Figure 19). These isolated natural products also having antibacterial activity against various strains [99].

### 4.3. Synthetic Molecules Containing an Indole Nucleus and Having Medicinal Importance

**Figure 20:**
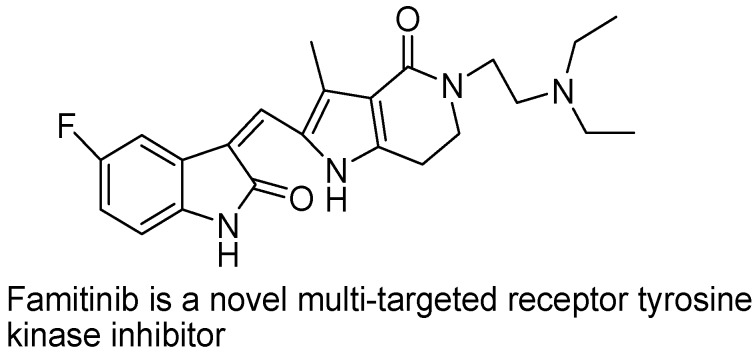
Famitinib.

**Figure 21:**
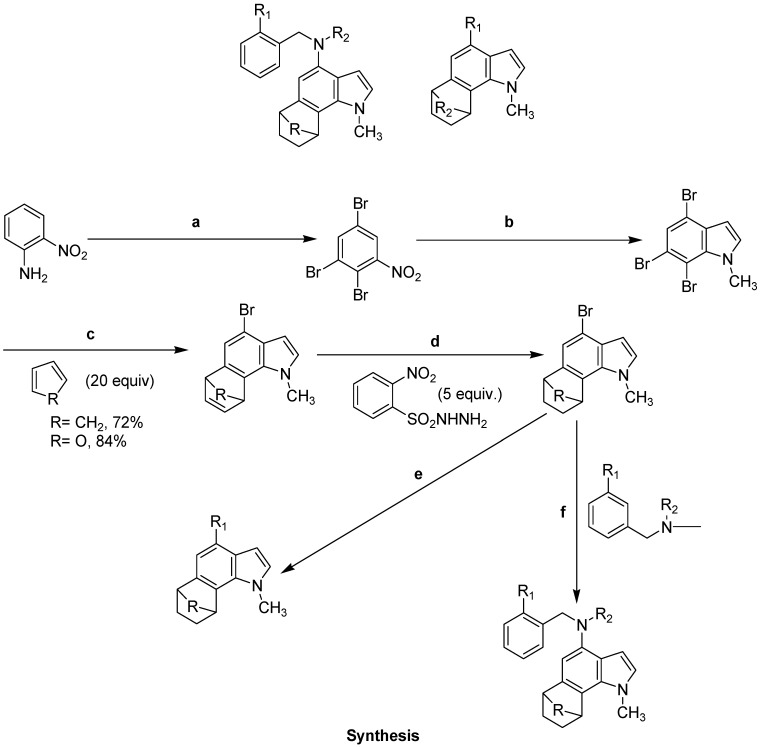
6,7-Annulated-4-substituted indoles.

Famitinib (Figure 20) is a novel multi-targeted receptor tyrosine kinase inhibitor under development for cancer treatment. Some studies have reported the metabolic and bioactivation pathways of famitinib. Researchers found that famitinib is well absorbed and extensively metabolized in cancer patients. Researchers found many metabolites of faminitib, some of which are important for drug activity by bioactivation of various enzymes and cells. A reactive metabolite of faminitib is N-desethylfaminitib (M3), whose steady-state exposure represented 7.2% to 7.5% that of the parent drug Antitumor effects of synthetic 6,7-annulated-4-substituted indole compounds reported in leukemic cells *in vitro* (Figure 21). Perchellet and his group synthesized sixty-six novel 6,7-annulated-4-substituted indole compounds, using a strategic combination of 6,7-indolyne cycloaddition and cross-coupling reactions under both Suzuki-Miyaura and Buchwald-Hartwig conditions, and found their effectiveness against murine L1210 leukemic tumor cell proliferation *in vitro* [100,101] These extensive metabolism or bioactivation processes may be helpful to establish the biological activity of drugs. Multiple enzymes, mainly CYP3A4/5 and CYP1A1/2, are involved in famitinib metabolic clearance [102].

**Figure 22:**
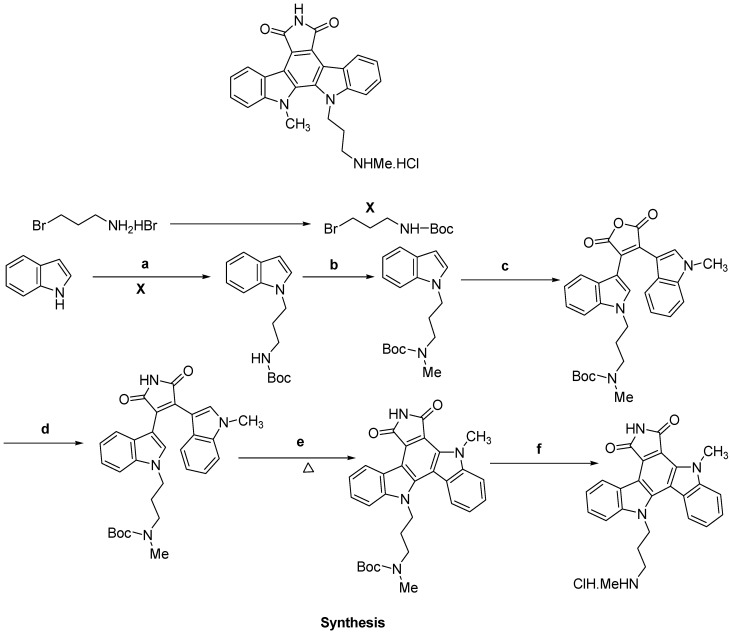
Bioactive indolocarbazole (ICP-125).

Researchers from the Gribble research group synthesized novel indolocarbazole derivatives and evaluated them biologically as novel checkpoint kinase 1 (Chk 1 having major role in cell cycle G1-S checkpoint) inhibitors (Figure 22). This group synthesized two nitrile analogues in the past, with variable lengths of the nitrile arm to investigate the effect of the nitrile chain-length on Chk1 activity. They found that some molecules induced DNA damage-induced cell cycle arrest. They also found that a three-carbon nitrile chain provided maximum activity. Bisindolylmaleimide was synthesized by subjected to the challenging oxidative cyclization reaction using palladium(II) trifluoroacetate and final step was deprotection of the Boc group. The final synthesized molecule, a potent bisindolylmaleimide, abrogates S phase arrest at 100 nM indicating that the compound is an inhibitor of Chk1. They also found that Pd(II) catalyzed oxidative cyclization is much more effective for bisindolylmaleimides bearing an amine group and found that a secondary amine or a nitrile are more desirable than a primary amine or amide on the chain [103].

*Reagents and conditions*: (**a**) Br_2_, MeOH/CH_2_Cl_2_ (1:1), 95% or *t*-BuONO, CuBr_2_, 75%; (**b**) CH_2_=CHMgBr (3 equiv.) THF, −40 °C, 45% or NaH, MEI, THF, 0 °C, 88%; (**c**) *n*-BuLi (1.2 eqiv) PhMe, −78 °C to RT; (**d**) Et_3_N, CH2Cl2, RT, 94%; (**e**) R_1_B(OH)_2_ (1.5 equiv.) Cs_2_CO_3_ (2 equiv.), Pd_2_ (dba)_3_ (2mol%) S-Phos (8 mol %) 1 Butanol 150 °C, MW (14–70%); (**f**) NaOt-amylate (2 equiv.), Pd_2_(dba)_3_ (2 mol%) X-Phoa (8 mol%) DME, 150 °C, MW, 78%

*Reagents and conditions*: (**a**) NaH, DMF; (**b**) NaH, THF or MeI; (**c**) (COCl)_2_, CH_2_Cl_2_ or Et_3_N, CH_2_Cl_2_, 1-methylindole-3-acetic acid; (**d**) HMDS, MeOH, DMF, 99%; (**e**) Pd (O_2_CCF_3_)_2_, DMF, 94%; (**f**) 1M HCl (Et_2_O), MeOH, 98%.

**Figure 23:**
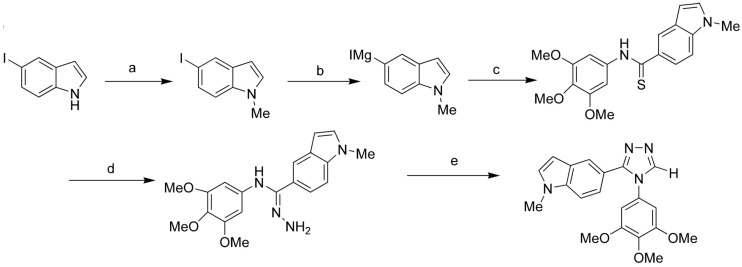
Synthesis of 1,2,4-triazoles with a *N*-methyl-5-indolyl moiety: tubulin inhibitor.

The Welsh group recently synthesized and biologically evaluated a series of novel tubulin polymerization inhibitors that contain a core indole and 1,2,4-triazole ring to retain the *cis* configuration required for bioactivity (Figure 23). These compounds exhibited potent tubulin polymerization inhibitory activity and cytotoxicity against a variety of cancer cells, including MDR cancer cell lines. Molecular docking and dynamics simulation were performed to study the inhibitor−protein interactions. Analysis of the inhibitor binding conformation in the colchicine binding site revealed specific residues that may play an important role in tubulin polymerization inhibitory activity and cytotoxicity [104].

*Reagents and conditions*: (**a**) (i) NaH, DMF, 0 °C; (ii) CH_3_I (**b**) *i*-PrMgCl, −10 °C; (**c**) 3,4,5-(CH_3_O)_3_C_6_H_2_NCS, −10 °C to rt; (**d**) NH_2_NH_2_, 0 °C to rt; (**e**) (CH_3_O)_3_CH, H_2_SO_4_(cat.), rt.

**Figure 24:**
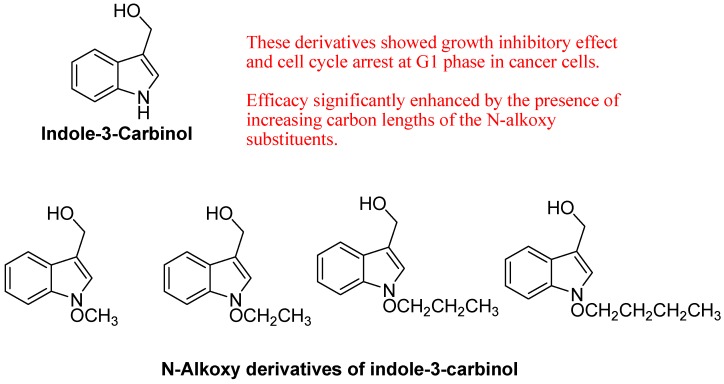
Synthetic derivatives of I3C.

Anti-proliferative response was investigated by Firestone group using synthetic I3C derivatives that contain substitutions at the indole nitrogen. Nitrogen substitutions included N-alkoxy substituents of one to four carbons in length, which inhibit dehydration and the formation of the reactive indolenine. Analysis of growth and cell cycle arrest of indole-treated human breast cancer cells elicited a striking increase in efficacy of the N-alkoxy I3C derivatives that is significantly enhanced by the presence of increasing carbon lengths of the N-alkoxy substituents. Compared to I3C, the half maximal growth arrest responses occurred at 23-fold lower indole concentration for N-methoxy I3C, 50-fold lower concentration for N-ethoxy I3C, 217-fold lower concentration for N-propoxy I3C, and 470-fold lower concentration for N-butoxy I3C. At these lower concentrations, each of the N-alkoxy substituted compounds induced the characteristic I3C response in that CDK6 gene expression, CDK6 promoter activity, and CDK2 specific enzymatic activity for its retinoblastoma protein substrate were strongly down regulated. 3-Methoxymethylindole and 3-ethoxymethylindole were approximately as bioactive as I3C, whereas both tryptophol and melatonin failed to induce the cell cycle arrest, showing the importance of the C-3 hydroxy methyl substituent on the indole ring. Their study implicates I3C-based N-alkoxy derivatives as a novel class of potentially more potent experimental therapeutics for breast cancer (Figure 24) [105].

**Figure 25:**
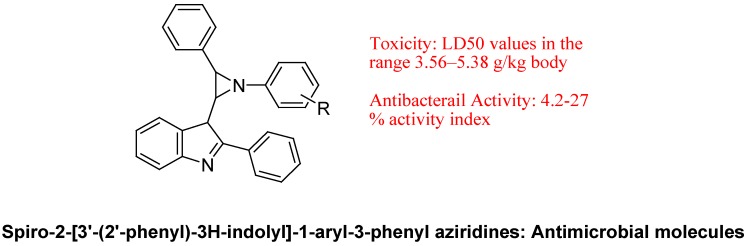
Bioactive spiro-2-[3′-(2′-phenyl)-3H-indolyl]-1-aryl-3-phenylaziridines.

Sharma *et al*. synthesized new series of bioactive spiro-2-[3′-(2′-phenyl)-3H-indolyl]-1-aryl-3-phenylaziridines (Figure 25) have been synthesized in quantitative yield via carbene insertion into C=N olefinic moiety of Schiff base. These compounds were found to exhibit excellent antibacterial activity against a series of Gram-positive and Gram-negative strains of bacteria. Furthermore, SAR studies elicited the key role of molecular refractivity and substituent nature/position on antibacterial properties. They found that there is the linear correlation between percentage activity index and molecular refractive index. Their results showed that steric parameters (MR) are highly correlated with biological activity. Electronic parameters come next in importance because the position of a substituent also has a substantial effect on the trend in biological activity. All synthesized compounds having less toxicity and LD_50_ values is in the range 3.56–5.38 g/kg body weight in rats [106].

3-(Nitromethylene)indolin-2-one analogues are valued protective agents against H_2_O_2_-induced apoptosis on cells, and for their cytotoxicity against the A549 and P388 lung cancer cell lines. A new synthesis method of 3-(nitromethylene)indolin-2-one analogues is reported, using the Henry reaction of isatin and N-substituted isatins with nitromethane followed by dehydration of the nitroaldol adduct with mesyl chloride. The use of diethylamine (rather than DBU) as the base catalyst in a solvent-free Henry reaction gave the nitroaldol adduct in sufficient purity as to allow its direct dehydration to nitroalkene [107]. 

**Figure 26:**
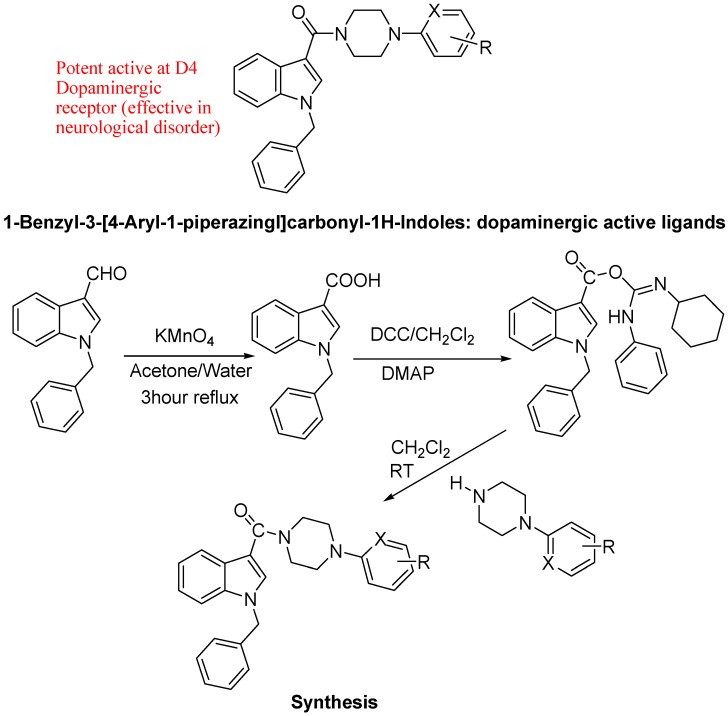
Functionalized 1-benzyl-3-[4-aryl-1-piperazingl]carbonyl-*1H*-indoles.

Recently functionalized 1-benzyl-3-[4-aryl-1-piperazingl]carbonyl-*1H*-indoles (Figure 26), were reported by the Pessoa-Mahana group as potential new class of bioactive ligands at D4 receptors. They synthesized these molecules in a five step sequence to provide the target indoleamides and yields were 75-92%. To the best of our knowledge, these are the first examples of indoleamides connected to arylpiperazines, which will be pharmacologically evaluated in the near future [108]. 

**Figure 27:**
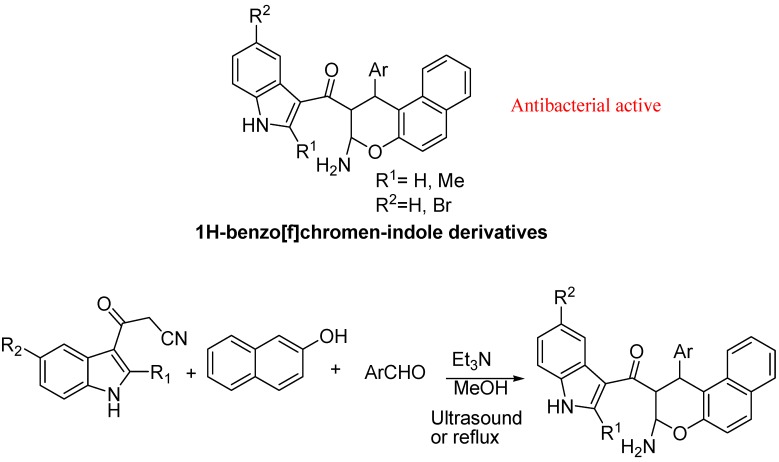
Functionalized indole-substituted chromene derivatives.

The Mamaghani group also developed a simple, efficient and versatile one-pot three-component protocol for the synthesis of novel derivatives of functionalized indole-substituted chromene derivatives (Figure 27) in a regiochemical manner by the reaction of 3-cyanoacetylindoles, β-naphthol, and arylaldehydes using triethylamine under ultrasonic irradiations and conventional conditions. The reaction induced by ultrasound offered better yields and much lower reaction times than the conventional heating. Most of the synthesized compounds exhibited excellent antibacterial activity against *Micrococcus luteus* [109].

**Figure 28:**
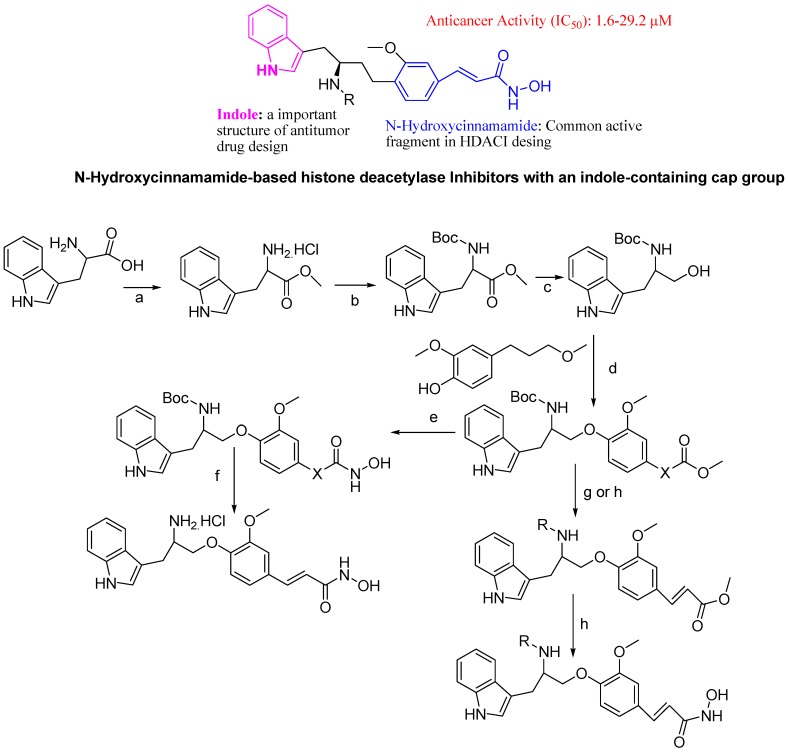
*N*-Hydroxycinnamamide-based histone deacetylase inhibitors.

More recently, comprehensive histone deacetylase inhibitors (HDACIs) structure-activity relationship (SAR) studies revealed that *N*-hydroxycinnamamide-based compounds were more stable than their straight chain analogues, and compounds having indole groups exhibited the best *in vivo* efficacy. Xu research group designed a novel series of *N*-hydroxycinnamamide-based HDACIs with an indole-containing cap group. Synthesis of these derivatives start with methyl ester protection and *tert*-butyloxycarbonyl (Boc) protection of l-tryptophan followed by LiAlH_4_ reduction. The first compound was prepared by methyl ester protection of ferulic acid and the second compound is prepared by hydrogenation of the first compound. Both the first and second compound were connected with an intermediate under Mitsunobu reaction conditions to get another 2 compounds, which were converted to corresponding hydroxamic acid compounds, respectively. Subsequent *N*-deprotection of the corresponding hydroxamic acid with trifluoroacetic acid (TFA) and subsequent amide condensation or sulfonylation gave intermediates which were treated with NH_2_OK (potassium hydroxylamine) to get target final active *N*-hydroxycinnamamide-based histone HDACIs compounds (Figure 28).

A novel series of N-hydroxycinnamamide-based histone HDACIs with an indole-containing cap group exhibited similar HDACs inhibition and *in vitro* antitumor potency to vorinostat (SAHA). Few synthesized molecule among them exhibited potent *in vitro* and *in vivo* antitumor activity [110].

**Figure 29:**
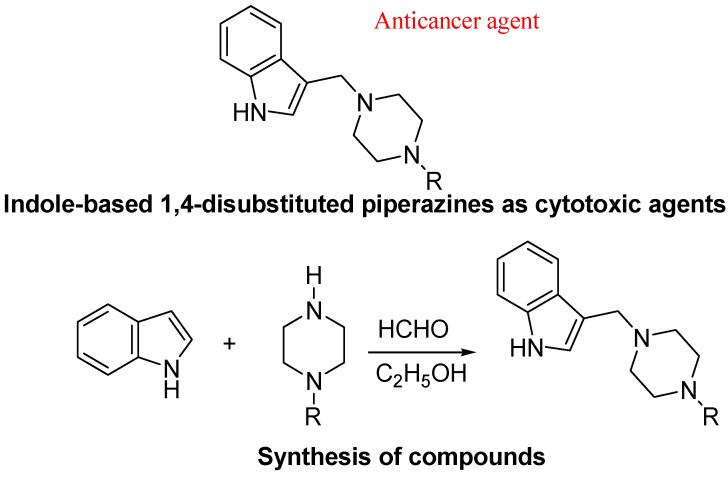
3-[(4-Substituted-piperazin-1-yl)methyl]-1*H*-indole derivatives.

A series of 3-[(4-substitutedpiperazin-1-yl)methyl]-1H-indole derivatives were synthesized via the Mannich reaction by Akkoc group. The cytotoxicity of compounds on 3 cell lines was studied and showed a variable extent of IC_50_ values. The cytotoxicity data of compounds demonstrate the importance of substitution at the N-4 position of piperazine (Figure 29). Compounds have an IC_50_ of less than 10 μM, which indicates significant cytotoxic activity [111].

**Figure 30:**
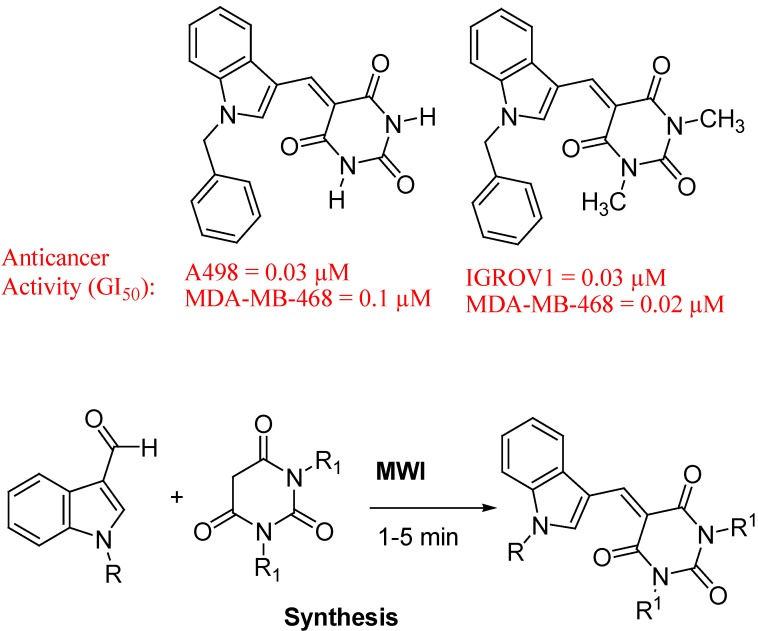
Hybrids of indole and barbituric acids.

Palwinder Singh *et al*. reported synthesis and anticancer activities of hybrids of indole and barbituric acids (Figure 30). By combining the structural features of indole and barbituric acid, new hybrid molecules were designed by Singh group and synthesized. They evaluated these molecules over 60 cell line panel of human cancer cells have identified two molecules with significant anticancer activities. Dockings study of two active molecules in the active sites of COX-2, thymidylate synthase and ribonucleotide reductase indicated their strong interactions with these enzymes [112].

**Figure 31:**
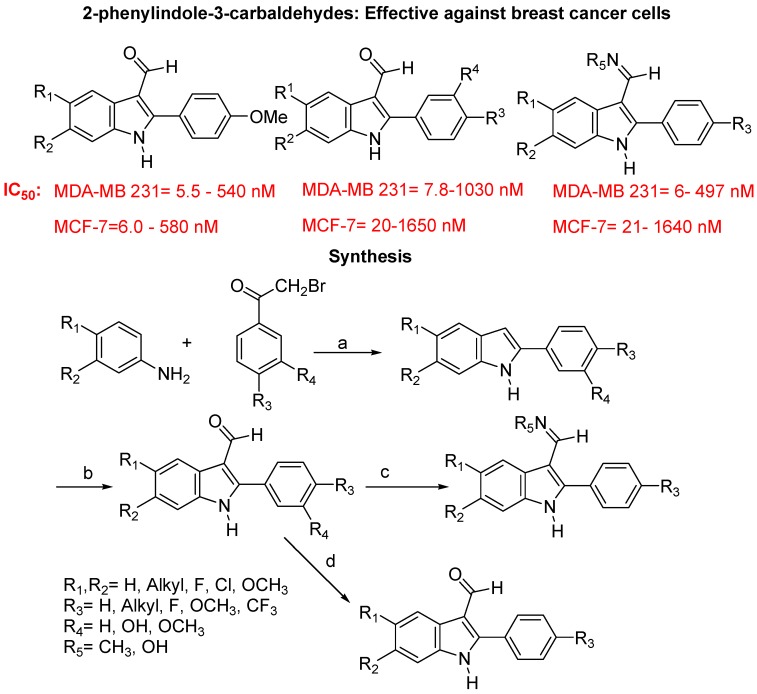
2-Phenylindole-3-carbaldehydes derivatives as anticancer agents.

Doris Kaufmann *et al*. synthesized antimitotic activities of 2-phenylindole-3-carbaldehyde in human breast cancer cells (Figure 31). This study revealed that the 2-phenylindole-3-carbaldehydes are an interesting class of compounds with high anti-proliferative activity in two breast cancer cell lines. They showed that tubulin is the primary target of synthesized molcules which inhibited the polymerization of tubulin to functional microtubules by binding to the colchicine binding site. This interaction with tubulin leads to cell cycle arrest in the G_2_/M phase and probably leads to an apoptotic cell death. 

*Reagent and conditions*: (a) SOCI_2_, CH_3_OH, 98%. (b) (Boc)_2_O, Et_3_N, DCM, 80%. (c) LiAIH_4_, anhydrous THF, 0 °C, 86%. (d) PPh_3_, DEAD, anhydrous THF, 0 °C, 75% for 8a, 79% for 8b. (e) NH_2_OK, CH_3_OH, 51% for 9a, 49% for 9b. (f) HCI, anhydrous EtOAc, 90%. (g) (i) TFA, Et_3_N, DCM; (ii) R'COOH, TBTU, Et_3_N, THF, 52–75% for two steps. (h) (i) TFA, Et_3_N, DCM; (ii) R'SO_2_CI, Et_3_N, DCM, 60–76% for two steps. (i) NH_2_OK, CH_3_OH, 31–54%.

The *in vitro* potencies of some of the aldehydes are in same range as those of vincristine and combretastatin A-4. Their preliminary investigations on the *in vivo* activity, however, showed that these aldehydes do not inhibit the growth of tumors. One of the possible reasons might be the instability of the aldehyde function toward metabolic reactions and insufficient bioavailability could be another reason. In order to overcome this problem they did modification of the carbonyl function to improve the metabolic stability of this essential structural element. Two of these modifications, conversion of the aldehydes to methyl imines and oximes, respectively, are included in their present study [113]. However, outcome of these modifications are not reported yet.

*Reagent and conditions*: (a) xylene, *N,N*-dimethylaniline, 170 °C; (b) POCl_3_, DMF, 15–70 °C, 3 h; (c) MeNH_2_, EtOH, 40 °C, 16 h; (d) H_3_NOH+Cl-, H_2_O/EtOH, NaOAc, reflux, 3 h.

**Figure 32:**
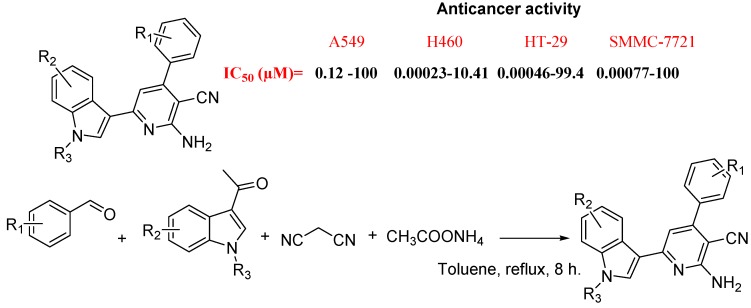
2-Amino-3-cyano-6-(1*H*-indol-3-yl)-4-phenylpyridine derivatives

Fan Zhang *et al*., synthesized and evaluated *in vitro* anti-tumor activity of 2-amino-3-cyano-6-(1*H*-indol-3-yl)-4-phenylpyridine derivatives (Figure 32). These derivatives screened for their cytotoxic activity against four human cell lines (A549, H460, HT-29 and SMMC-7721) and displayed excellent anti-tumor activity against these cell lines. Their pharmacological data indicated that introduction of indole core improved the anti-tumor activity of the 4,6-diaryl-2-amino-3-cyanopyridines. From structure activity relationships, they concluded that introduction of methyl group to the 1-postion of indole slightly enhanced the cytotoxic activity, while halogen or no substituent on indole ring was favorable. They also confirmed that introduction of halogen groups into benzene ring was essential for their cytotoxic activity and the 3-bromo-4,5-dimethoxy group was the best of the tested 3,4,5-trisubstituted groups [114]. 

**Figure 33:**
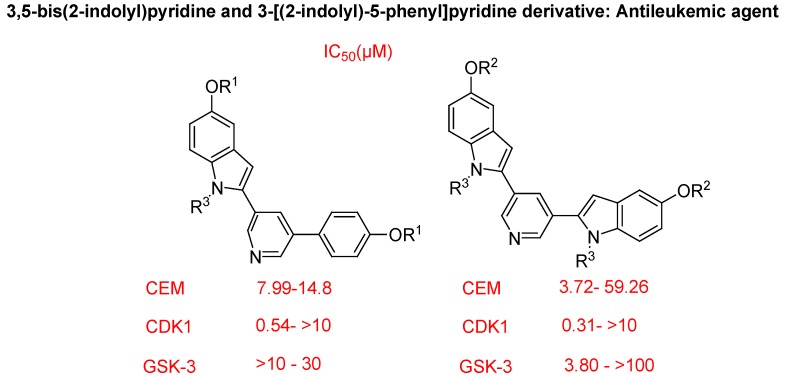
Mono- and bis-indole-pyridine derivatives.

Ulrich Jacquemard *et al*. synthesized a novel series of mono- and bis-indole-pyridine derivatives as CDK inhibitors and cytotoxic agents (Figure 33). Concerning their mechanism of action, they reached two major conclusions. First, a number of DNA-binding ligands were identified, in particular those bearing one or two cationic side chains. In terms of DNA recognition, the most interesting molecule behaves as a conventional DNA minor groove binder. Second, they identified and characterized three CDK1 inhibitors, which exhibit selectivity over GSK-3 and these compounds can fit into the ATP pocket of the enzyme according to docking study [115].

**Figure 34:**
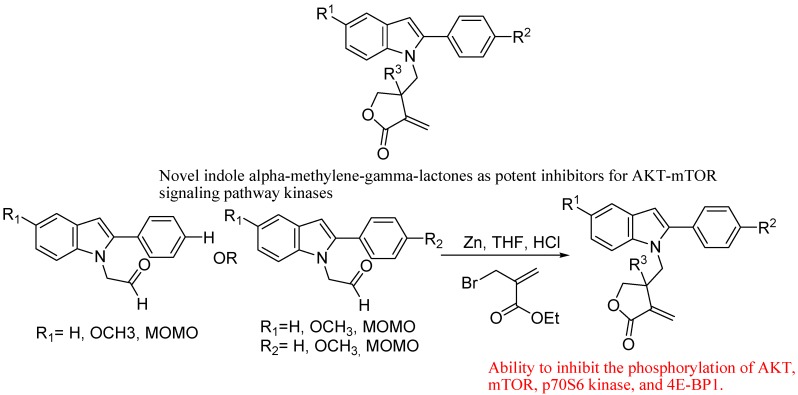
Indole α-methylene-γ-lactones.

Nassar *et al*. reported (*in vitro*) antitumor and antimicrobial activity of some pyrazoline, pyridine, and pyrimidine derivatives linked to an indole moiety. They reported that the aldol condensation reaction between 3-indolaldehyde and 4-methoxyacetophenone gave a chalcone compound from which some pyrazoline, pyridine, and pyrimidine derivatives linked to the indole moiety were obtained and found to have promising antitumor and antimicrobial activities [116]. Ding *et al*. reported novel indole α-methylene-γ-lactones as potent inhibitors for AKT-m TOR signaling pathway kinases (Figure 34). Their results indicated that keeping the γ-position of the lactone not substituted is crucial for the inhibition activity. Besides, a methoxy substituent on the phenyl is more favorable than on the indole ring [117].

**Figure 35:**
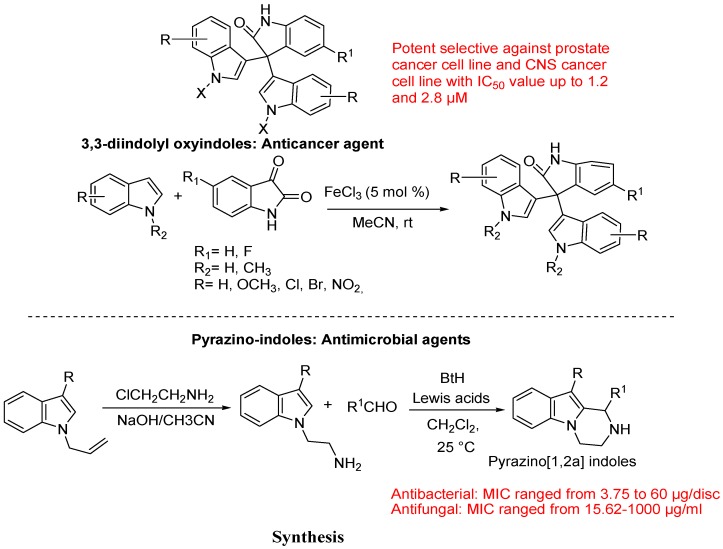
3,3-Diindolyl oxyindoles and pyrazino[1,2α]indoles.

Recently, Ahmed Kamal *et al*. reported synthesized of 3,3-diindolyl oxyindoles efficiently catalysed by FeCl_3_ and their *in vitro* evaluation for anticancer activity (Figure 35). 

They developed a simple and highly efficient method for the conversion of 3,3-diindolyl oxyindoles from indole and isatin using 5 mol% of FeCl_3_ in high yields with various advantage over previous reported methods. Reported compounds exhibited potential anticancer potency thereby suggesting that these scaffolds as possible anticancer agents by the structural modification in both indole and oxyindole moieties for improving the anticancer efficacy [118].

The Verma group described the synthesis and antimicrobial activity of substituted 1,2,3,4-tetrahydropyrazino[1,2-*a*]indoles as potential candidates for antimicrobial activity against various microbial strains having less toxicity [119,120,121]. Synthesized compounds have antibacterial activity against pathogenic strains of S. aureus (MTCCB 737), S. typhi (MTCCB 733), P.aeruginosa(MTCCB 741), S. thermonitrificans (MTCCB 1824) and E. coli (MTCCB 1652). They also examined antifungal activity of the synthesized molecules against pathogenic strains of Aspergillus fumigates (ITCC 4517), Aspergillus flavus (ITCC 5192) Aspergillus niger (ITCC 5405) and Candida albicans (ITCC No 4718). They used benzotriazole for the synthesis of subsitituted for the synthesis of subsitituted-1,2,3,4-tetrahydropyrazino[1,2-*a*]indoles. 1-(2-Aminoethyl)indoles were obtained by the reaction of indole or 3-methyl indole with 2-chloroethylamine hydrochloride. 1-Substituted-1,2,3,4-tetrahydro-pyrazino[1,2-*a*]indoles were obtained as racemic mixtures in high yields by the reaction of 2-(3-methyl-1*H*-indol-1-yl)ethylamine with benzotriazole and aldehydes in the presence of a catalytic amount of Lewis acid (AlCl_3_, ZnCl_2_, ZnBr_2_) or protic acid (CH_3_SO_3_H) at 25 °C indichloromethane (Figure 35).

**Figure 36:**
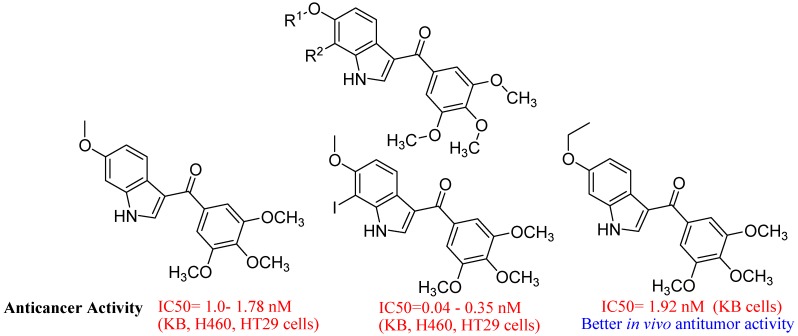
3-Aroylindoles.

Yu-Shan Wu *et al.* reported synthesis and evaluation of 3-aroylindoles as anticancer agents (Figure 36). They evaluated hydroxylated and O-demethylated phase I metabolites of potent antitumor agent 6-methoxy-3-(3′,4′,5′-trimethoxy-benzoyl)-1H-indole. They found that four of five metabolites are active against various cancer cell lines in the nanomolar concentration range. The iodo derivative of the most potent 7-hydroxy metabolite, exhibited extremely potent anticancer activity against cancer cells, with the IC_50_ reaching picomolar potency in the KB, H460, and HT-29 cell lines. Further structure−activity relationship studies at the seventh positions of the indole may provide new insights into combretastatin analogue design in the future [122]. 

**Figure 37:**
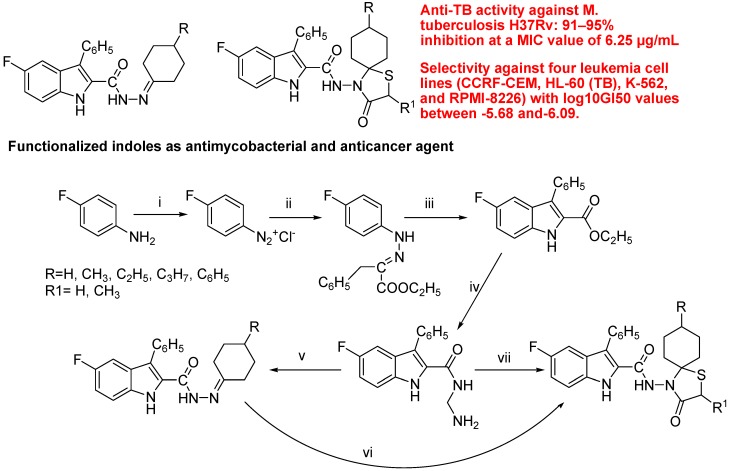
Synthesis of functionalized indoles.

Cihan-Üstündağ *et al*., reported synthesis and evaluation of functionalized indoles as antimycobacterial and anticancer agents (Figure 37). In the search for effective and selective antitubercular and anticancer agents, they synthesized novel hydrazone and spirothiazolidinone derivatives of the 5-fluoro-3-phenyl-1H-indole scaffold. They evaluated all componds for *in vitro* anti-TB activity against M. tuberculosis H37Rv. The spirothiazolidinone derivatives bearing a methyl or propyl group at position 8 of the spiro ring, were the most active compounds showing 91–95% inhibition at a MIC value of 6.25 µg/mL. The antitumor screening of compounds against 60 different cell lines revealed moderate to good anti-proliferative activity. They suggested that indolylspirothiazolidinones may be considered as interesting and encouraging pharmacophores for antimycobacterial and anticancer drug discovery [123]. 

*Reagents and conditions*: (i) 7% NaNO_2_, EtOH, conc. HCl, 0 °C; (ii) ethyl 2-benzyl-3-oxo-butanoate, KOH, EtOH, 0 °C; (iii) conc. HCl, reflux, 4 h; (iv) H_2_NNH_2_.H_2_O, EtOH, reflux, 6h; (v) 4-(non)substituted cyclohexanone, abs. EtOH, reflux, 5–6 h; (vi) mercaptoacetic acid/2-mercaptopropionic acid, dry benzene, reflux, 5–6 h; (vii) 4-(non)substituted cyclohexanone, mercaptoacetic acid/ 2-mercaptopropionic acid, dry benzene, reflux, 5–6 h.

**Figure 38:**
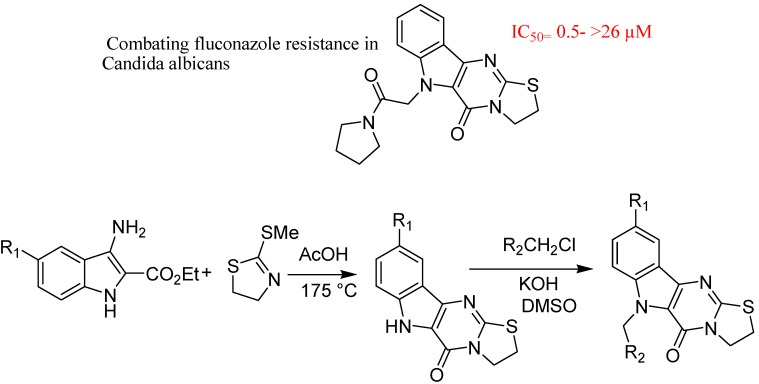
Tetracyclic indoles.

Youngsaye *et al*. reported overcoming of fluconazole resistance in *Candida albicans* clinical isolates with tetracyclic indoles (Figure 38). They reported SAR studies on the structurally distinct compound that was also identified as a chemosensitizer of *C. albicans* in the primary screen. The resulting small-molecule probe ML229, along with ML189, should be useful tools for interrogating the molecular mechanism by which *C. albicans* acquires resistance against azole antifungals. To assist such efforts, they registered tetracyclic indole with the NIH Molecular Libraries Program as probe ML229 and it is available upon request [124]. 

**Figure 39:**
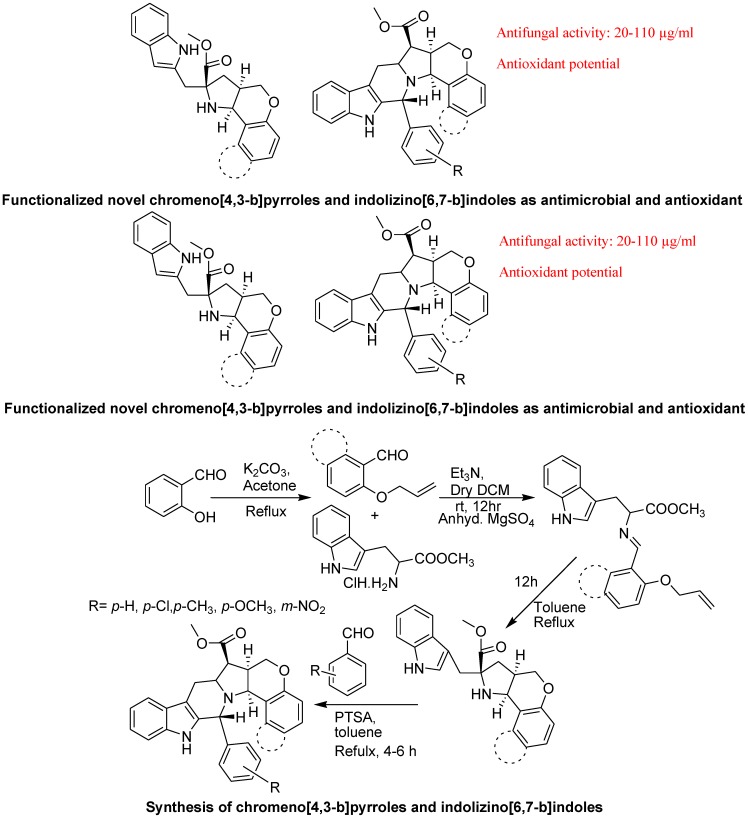
Chromeno[4,3-b]pyrroles and indolizino[6,7-b]indoles.

Arumugam *et al*. reported synthesis of highly functionalized novel chromeno[4,3-b]pyrroles and indolizino[6,7-b]indoles as potent antimicrobial and antioxidant agents (Figure 39). They synthesized a series of novel chromeno[4,3-b]pyrroles and indolizino[6,7-b]indoles by sequential intramolecular 1,3-dipolar cycloaddition and subsequent Pictet-Spengler cyclization. Four leads compounds displayed potent activity against four selected bacterial pathogens and two compounds exhibited good activity against two fungal organisms. Some compounds showed good antioxidant potential. They also demonstrated quantitative structure-activity relationship (QSAR) that confirmed indolizino[6,7-b]indole with electron withdrawing groups (–NO_2_, –Cl) attached directly to the phenyl ring were essential for activity [125].

**Figure 40:**
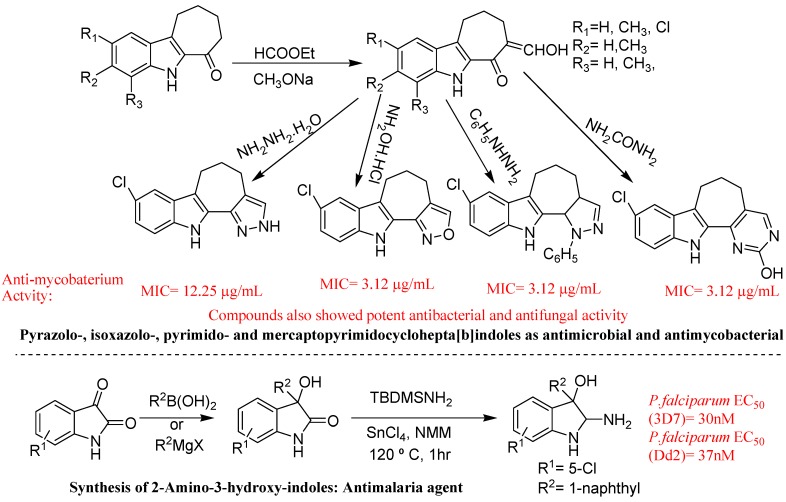
Cyclohepta[b]indoles and 2-amino-3-hydroxyindoles.

Yamuna *et al*. described synthesis, antimicrobial, antimycobacterial and structure-activity relationship of substituted pyrazolo-, isoxazolo-, pyrimido- and mercaptopyrimidocyclohepta[b]indoles (Figure 40). They prepared heterocyclic pyrazolo-, isoxazolo-, pyrimido-, and mercaptopyrimidocyclo hepta[b]indoles from 7-hydroxymethylene-7,8,9,10-tetrahydrocyclohepta[b]indol-6(5H)-ones by cyclocondensation with appropriate nucleophiles. They observed maximum activity in compounds having a chloro substituent in the cyclohepta[*b*]indole moiety. All these new cyclohepta[*b*]indole analogues were evaluated for their *in vitro* antimycobacterial activity against M. tuberculosis H37Rv (MTB) by the resazurin microtitre assay (REMA). Bioavailability and initial toxicity tests of the compounds indicated that the compounds have properties that make them suitable for further testing as potential drug candidates [126].

Diana *et al*. reported synthesis and antitumor activity of 3-(2-phenyl-1,3-thiazol-4-yl)-1*H*-indoles and 3-(2-phenyl-1,3-thiazol-4-yl)-1*H*-7-azaindoles. Based on a 2,4-bis(3′-indolyl)imidazole skeleton, two new series of phenylthiazolylindoles and phenylthiazolyl-7 azaindoles were obtained by Hantzsch reaction between substituted phenylthioamides and the α bromoacetyl derivatives. They tested some derivatives at the National Cancer Institute against a panel of 60 tumor cell lines derived from nine human cancer cell types, showed inhibitory effects against all cell lines investigated at micromolar to nanomolar concentrations. They found two molecules exhibited a high affinity for CDK1, with IC_50_ values of 0.41 and 0.85 μM [127]. 

Urgaonkar *et al*. described the potent *in vivo* anti-malaria activity of 2-amino-3-hydroxyindoles (Figure 40). They identified 2-amino-3-hydroxy-indoles as a novel chemical class with potent *in vitro* and *in vivo* antimalaria activity. They have developed a concise synthetic strategy to efficiently synthesize analogues in quantities sufficient for medicinal chemistry exploration. This method establishes the unprecedented use of TBDMSNH_2_ as an ammonia surrogate and allows for the first enantioselective synthesis of 2-amino-3-hydroxy-indoles [128].

**Figure 41:**
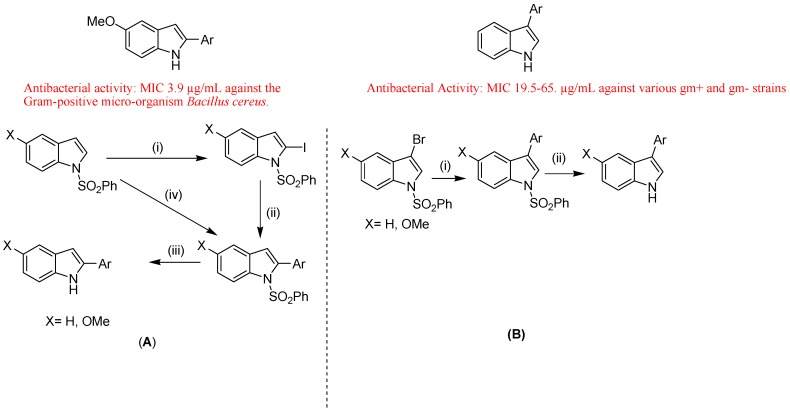
2- and 3-Arylindoles.

Leboho *et al.* reported synthesis of 2- and 3-arylindoles (Figure 41) and 1,3,4,5-tetrahydropyrano[4,3-b]indoles and their antibacterial and antifungal activity. They synthesized 2-aryl indoles from the 1-(phenylsulfonyl)indole derivatives using magnesiation followed by iodination, and 2-iodinated compounds were subjected to Suzuki-Miyaura reactions. In addition, they made the 3-arylindoles from the corresponding 3-bromoindoles using Suzuki-Miyaura reactions. They prepared 1,3,4,5-tetrahydropyrano[4,3-b]indoles from 1-(phenylsulfonyl)indole by magnesiation followed by treatment with allylbromide. The product was converted into [2-allyl-1-(phenylsulfonyl)-1*H*-indol-3-yl]methanol which upon exposure to Hg(OAc)_2_ and NaBH_4_ afforded tetrahydropyrano[4,3-b]indoles. These synthesized 2- and 3-arylindoles displayed potent antimicrobial activity, against the Gram-positive micro-organism *Bacillus cereus* [129]. Giraud *et al*. designed and evaluated 3-(imidazol-1-ylmethyl)indoles as antileishmanial agents. They synthesized a new series of 1-benzyl-3-(imidazol-1-ylmethyl) indoles according to a 3D-QSAR predictive model and assayed for their antiparasitic activity upon *Leishmania mexicana* promastigotes. They showed IC_50_ values of these molecules ranging from 2.3 to 32 μM and this mainly illustrated the importance of the hydrophobic parameter the *para*-position of the benzyl group. They used a carig diagram to select original electro-donating and lipophilic substituents, in order to improve the activities of these compounds and to check the potential influence of the electronic parameter on this particular position. They confirmed that only the hydrophobic field is essential for a high level of activity of new compounds (IC_50_ between 2.5 and 5.4 μM) [130]. H. Xu *et al*. described developments of indoles as anti-HIV-1 inhibitors. AIDS has always been a global health threat and the leading cause of deaths due to the rapid emergence of drug-resistance and unwanted metabolic side effects. They reported that indole derivatives have been considered as one class of promising HIV-1 inhibitors, such as delavirdine approved by the Food and Drug Administration (FDA) in 1997 for use in combination with other antiretrovirals in adults with HIV infection. They made focus on the synthesis and anti-HIV-1 activity of indole derivatives, with the structure-activity relationship (SAR) for some derivatives [131].

*Reagents and Conditions*: (**A**)(i) iPrMgCl, (iPr)_2_NH, THF, I_2_, X = H, 79%, X = OMe, 89%; (ii) 10% Pd(PPh_3_)_4_, DME/EtOH, aq Na_2_CO_3_, Aryl boronic acid, reflux; (iii) K_2_CO_3_, MeOH; (iv) (a) iPrMgCl, (iPr)_2_NH, THF; (b) 5% Pd(PPh_3_)_4_, 2 equiv 1,4-dibromo-2,5-dimethoxybenzene. (**B**) (i) 10% Pd(PPh_3_)_4_, DME/EtOH, aq Na_2_CO_3_, aryl boronic acid, reflux; (ii) K_2_CO_3_, MeOH.

**Figure 42:**
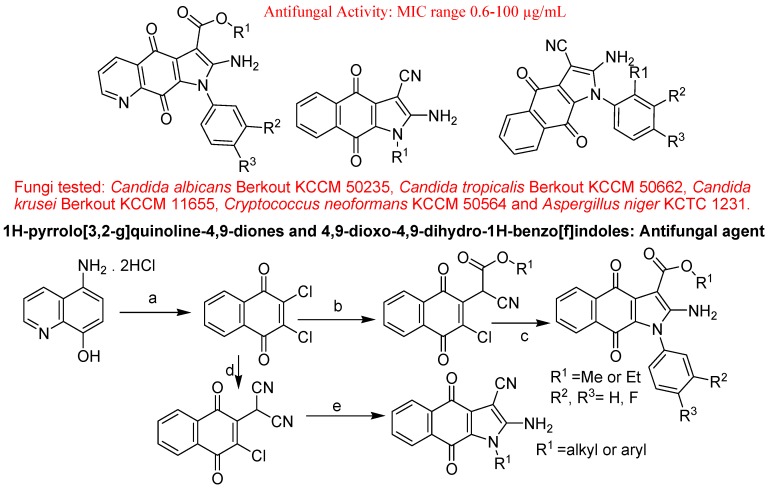
1*H*-Pyrrolo[3,2-g]quinoline-4,9-diones and 4,9-dioxo-4,9-dihydro-1*H*-benzo[f]indoles.

Ryu *et al*. described synthesis and antifungal activity of 1H-pyrrolo[3,2-g]quinoline-4,9- diones and 4,9-dioxo-4,9-dihydro-1H-benzo[f]indoles (Figure 42). Alkyl-2-(7-chloro-5,8-dioxo-5,8-dihydroquinolin-6-yl)-2-cyanoacetate was synthesized by nucleophilic substitution of 6,7-dichloro quinoline-5,8-dione with a dihydroquinolin-6-yl)-2-cyanoacetate equivalent of alkyl cyanoacetate in the presence of NH_4_OH. 1*H*-Pyrrolo[3,2-g]quinoline-4,9-diones and 4,9-dioxo-4,9-dihydro-1*H*- benzo[f]indoles were synthesized by cyclization of compounds with amines in EtOH. Some synthesized compounds showed potent antifungal activity against pathogenic fungi [132].

*Reagents and conditions*: (a) NaClO_3_/HCl/H_2_O/60° C/30 min; (b) methyl cyanoacetate or ethyl cyanoacetate/EtOH/NH_4_OH/rt/10 min (c) arylamine/EtOH/reflux/5 h; (d) malononitrile/triethyl-amine/EtOH/reflux/20 min; (e) alkyl or arylamine/EtOH/reflux/5 h.

## 5. Conclusions

Indole derivatives are very important heterocyclic compounds in the drug-discovery studies. They represent a very important class of molecules that play a major role in cell biology and are potential naturally occurring products. There has been an increasing interest in the use of indole derivatives as bioactive molecules against microbes, cancer cells, and various kinds of disorder in the human body. This paper reviews the current status and the recent studies of biologically important indole derivatives. The review is meant to present a general overview of the various research activities in this expanding field.
